# Global patterns and drivers of phylogenetic structure in island floras

**DOI:** 10.1038/srep12213

**Published:** 2015-07-22

**Authors:** Patrick Weigelt, W. Daniel Kissling, Yael Kisel, Susanne A. Fritz, Dirk Nikolaus Karger, Michael Kessler, Samuli Lehtonen, Jens-Christian Svenning, Holger Kreft

**Affiliations:** 1 Biodiversity, Macroecology & Conservation Biogeography Group, University of Göttingen, 37077 Göttingen, Germany; 2 Systemic Conservation Biology, University of Göttingen, 37073 Göttingen, Germany; 3 Institute for Biodiversity and Ecosystem Dynamics (IBED), University of Amsterdam, 1090 GE Amsterdam, The Netherlands; 4 Biodiversity and Climate Research Centre (BiK-F) & Senckenberg Gesellschaft für Naturforschung, 60325 Frankfurt, Germany; 5 Institute of Systematic Botany, University of Zurich, 8008 Zurich, Switzerland; 6 Department of Biology, University of Turku, 20014 Turku, Finland; 7 Section for Ecoinformatics & Biodiversity, Department of Bioscience, Aarhus University, 8000 Aarhus C, Denmark

## Abstract

Islands are ideal for investigating processes that shape species assemblages because they are isolated and have discrete boundaries. Quantifying phylogenetic assemblage structure allows inferences about these processes, in particular dispersal, environmental filtering and *in-situ* speciation. Here, we link phylogenetic assemblage structure to island characteristics across 393 islands worldwide and 37,041 vascular plant species (representing angiosperms overall, palms and ferns). Physical and bioclimatic factors, especially those impeding colonization and promoting speciation, explained more variation in phylogenetic structure of angiosperms overall (49%) and palms (52%) than of ferns (18%). The relationships showed different or contrasting trends among these major plant groups, consistent with their dispersal- and speciation-related traits and climatic adaptations. Phylogenetic diversity was negatively related to isolation for palms, but unexpectedly it was positively related to isolation for angiosperms overall. This indicates strong dispersal filtering for the predominantly large-seeded, animal-dispersed palm family whereas colonization from biogeographically distinct source pools on remote islands likely drives the phylogenetic structure of angiosperm floras. We show that signatures of dispersal limitation, environmental filtering and *in-situ* speciation differ markedly among taxonomic groups on islands, which sheds light on the origin of insular plant diversity.

Global patterns of plant diversity and their links with environmental variables are increasingly well documented^1^, but our understanding of the underlying processes generating these patterns, including speciation and extinction, dispersal, and environmental filtering, lags behind^2^. A promising way forward is to incorporate phylogenetic data to add a long-term evolutionary perspective to biodiversity research^2^,^3^,^4^. Community ecologists increasingly use phylogenetic information to infer assembly processes^5^,^6^, because these data add a temporal dimension that allows improved inference about the processes generating and maintaining diversity patterns^4^. However, studies of phylogenetic patterns at macro-scales have so far mostly been descriptive and focused on terrestrial vertebrates (e.g. ref. 7). Disentangling the processes generating patterns of phylogenetic diversity at larger spatial scales is therefore at the forefront of macroecological and macroevolutionary research^8^,^9^.

Islands are ideal study systems for testing the roles of dispersal, environment and *in-situ* speciation in shaping diversity patterns in general and the phylogenetic structure of species assemblages in particular^4^,^10^,^11^. Due to their isolated nature, islands are characterized by limited colonization and evolutionarily unique biotas^12^,^13^. However, most broad-scale studies of island biodiversity have focused on species richness (e.g. ref. 14, but see ref. 15 for turnover), and studies of phylogenetic structure on islands have so far focused on lineages with low insular diversity, little *in-situ* speciation and low ability to colonize islands (e.g. mammals in^10^, snakes^16^). Nonetheless, there is mounting evidence that islands show distinct patterns of phylogenetic structure compared to mainlands^7^,^9^,^10^,^16^.

The roles of dispersal, environmental filtering and *in-situ* speciation in shaping the phylogenetic structure of island floras at a global scale can be addressed by assessing relationships between physical and bioclimatic island characteristics^17^ and phylogenetic community metrics^6^,^18^ (Fig. 1). Whereas the expected phylogenetic patterns can be similar for the three focal processes, the expected physical and bioclimatic correlates should differ between them and allow distinguishing the different processes. For instance, dispersal filtering refers to the imbalanced colonization of islands due to missing adaptations or lack of opportunities for long-distance dispersal in some species^13^ (Fig. 1). If dispersal-related traits show a strong phylogenetic signal (i.e. closely related species share similar dispersal abilities^3^,^19^; Supplementary Text S1), then dispersal filtering should lead to phylogenetically clustered island assemblages^20^. This should be true no matter whether phylogenetic structure is compared to the mainland species pool (indicating the initial dispersal filter of becoming an island lineage) or whether phylogenetic structure of an island is compared to a pool of island species (indicating the dispersal filtering of lineages pre-adapted to colonization for a particular island). The number of past immigration events should be lowest on isolated islands that were not connected to the mainland or other islands in the past^21^. The strength of the dispersal filter and its effect on the phylogenetic structure of island assemblages should hence be related to island isolation and geologic history (e.g. continental vs. oceanic islands), and the size and composition of the mainland species pool (Fig. 1).

Beyond dispersal filtering, environmental filtering plays a key role in structuring island floras because only a subset of arriving species can tolerate the environmental conditions of each island^13^ (Fig. 1). Environmental filtering should lead to phylogenetically clustered island assemblages if adaptations to environments are not randomly distributed over the phylogeny of island species^3^,^22^ (Supplementary Text S1). Environmental heterogeneity and past and present climate have been identified as key drivers of species richness on islands^9^,^14^,^23^,^24^ and we therefore expect them to be key environmental filters that differ from those correlates of phylogenetic clustering that indicate dispersal filtering (Fig. 1). We predict the strength of environmental filtering to decrease with increasing environmental heterogeneity and to be least pronounced for warm and humid conditions under which most lineages originated (tropical niche conservatism^25^,^26^).

Finally, *in-situ* speciation creates clusters of closely related species^4^, also leading to low phylogenetic diversity relative to species richness and high levels of phylogenetic clustering^27^ (Fig. 1). Again, several island characteristics that partly differ from those influencing dispersal and environmental filtering are known to affect the probability of *in-situ* speciation and as a result should correlate with phylogenetic structure. First, island isolation increases the probability of *in-situ* speciation because isolation reduces gene flow with continental source populations, allowing island populations to evolve independently^28^. Second, the probability of *in-situ* speciation increases with island area and environmental heterogeneity, as these increase the probability of intra-island reproductive isolation and promote ecological speciation through specialization and adaptive radiations^24^,^29^. Finally, island geologic history and age are expected to influence rates of *in-situ* speciation and phylogenetic structure. Whereas continental fragments and shelf islands harbour relatively saturated biota from their formation onwards, volcanic islands or islands emerging from uplifted seafloor initially provide open arenas for new species to establish, and thus foster high rates of speciation^11^,^30^. The effects of *in-situ* speciation on the phylogenetic structure of island floras should hence be related to island isolation, area, heterogeneity, geologic history and age.

Here, we test the framework outlined above by investigating the effects of abiotic island characteristics related to dispersal, environmental filtering and speciation on the phylogenetic structure of 393 island floras worldwide using dated phylogenies and a unique data set of 118,062 island occurrences of 37,041 vascular plant species. We compare patterns and predictors of phylogenetic structure for three plant groups that show different dispersal- and speciation-related traits and adaptations to climate (Supplementary Text S1). Specifically, we contrast angiosperms and ferns using family-level phylogenies^31^,^32^,^33^, and for more detailed insights, we contrast palms and ferns using genus-level phylogenies^33^,^34^. We relate phylogenetic diversity (measured as standardized effect size (PD_es_) of Faith’s phylogenetic diversity (PD)^18^) and the degree of phylogenetic clustering vs. overdispersion (i.e. higher vs. lower relatedness than expected by chance, measured as standardized effect size (MPD_es_) of mean pairwise phylogenetic distances (MPD)^6^) of the three plant groups to environmental island characteristics to test four non-mutually exclusive hypotheses (Fig. 1). **H1 (group differences)**: global patterns and determinants of phylogenetic structure vary among taxonomic groups due to differences in their dispersal ability, climatic adaptations, distribution patterns and diversification rates (Supplementary Text S1). For example, we expect dispersal- and speciation-related environmental predictors to have less influence on phylogenetic structure for ferns than for angiosperms overall and palms, due to a higher dispersal ability. **H2 (dispersal filtering)**: phylogenetic diversity and overdispersion increase with factors that facilitate dispersal to islands (e.g. low island isolation). **H3 (environmental filtering)**: phylogenetic diversity and overdispersion are higher if island environmental conditions fit bioclimatic requirements of a clade (e.g. tropical climate for palms). **H4 (*****in-situ*** **speciation)**: factors that increase the probability of speciation on islands (e.g. larger island area) are negatively related to phylogenetic overdispersion.

## Results

### Patterns of phylogenetic structure

Angiosperms occurred on 365 of 375 islands (97%), palms on 170 of 386 islands (44%) and ferns on 255 of 328 islands (78%; Fig. 2). Within each of the three plant groups, log_10_-transformed PD and species richness were strongly and positively related to each other (Fig. 2; Supplementary Table S1; all Pearson r > 0.83, p < 0.001). Phylogenetic metrics accounting for species richness (PD_es_ and MPD_es_^5^,^6^) were either not correlated (ferns, angiosperm MPD_es_) or negatively correlated (palms, angiosperm PD_es_; −0.36 > r >−0.59, p < 0.01) with species richness (Supplementary Table S1).

Measures of phylogenetic structure in angiosperms and palms were mostly negative (PD_es_: 93% of all islands for angiosperms, 73% for palms; MPD_es_: 69% for angiosperms, 80% for palms), i.e. PD and MPD were mostly smaller than expected by chance, indicating phylogenetic clustering (Fig. 3). Ferns showed less variation in phylogenetic community metrics and a greater proportion of positive PD_es_ (59%) and MPD_es_ values (66%; Fig. 3), indicating phylogenetic overdispersion. PD_es_ and MPD_es_ were strongly correlated within palms and ferns (r > 0.84, p < 0.001), but less so within angiosperms (r = 0.56, p < 0.01; Supplementary Table S1); some continental fragments showed high angiosperm PD_es_ and at the same time low MPD_es_ (Fig. 3).

PD_es_ values of the three taxonomic groups were not correlated with each other (Supplementary Table S2). MPD_es_ values were moderately correlated for angiosperms and palms (r = 0.40, p < 0.01) as well as angiosperms and ferns (r = 0.30, p < 0.01). Family- and genus level metrics were strongly correlated for ferns (r > 0.91, p < 0.001).

### Relationships with environmental predictors

**Figure 1:**
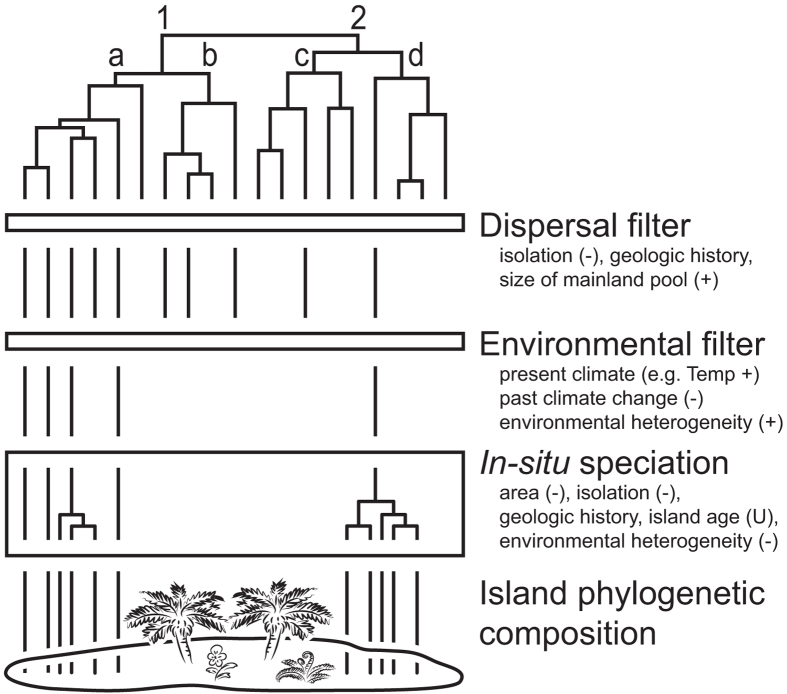
General framework for testing hypothesized effects of dispersal filters, environmental filters and *in-situ* speciation on the phylogenetic structure of island assemblages. Clade 1 represents good dispersers, clade 2 weak dispersers. Clades *a* and *d* share adaptations to environmental conditions on islands, clades *b* and *c* do not. If dispersal-related traits and environmental adaptations are not randomly distributed over the phylogeny, then dispersal and environmental filters should increase the probability of island colonization in certain clades and increase phylogenetic clustering of the island assemblage. Although such phylogenetic clustering on young islands is initially mostly observed relative to the mainland species pool, radiations within island lineages and extinction due to environmental changes should further increase phylogenetic clustering in some islands relative to a pool of island species. The strength of dispersal and environmental filters and the probability of *in-situ* speciation on islands should be related to the listed physical, geologic and bioclimatic island characteristics. Symbols beside environmental variables (not indicated for categorical variables) indicate the hypothesized relationships with the standardized effect sizes of phylogenetic diversity (PD_es_) and mean pairwise phylogenetic distance (MPD_es_): – negative, + positive, U U-shaped.

We used predictor variables describing the environmental conditions on islands and multi-predictor Generalized Additive Models (GAMs) to test for the effects of dispersal, environmental filtering and *in-situ* speciation on the phylogenetic structure of island floras (Table 1, Fig. 1). The best models explained up to 49% of variation in PD_es_ for angiosperms and 52% for palms, but only 15% (family-level) and 18% (genus-level) for ferns (Supplementary Table S3). When regional biogeographic history was considered in models by including Takhtajan’s floristic subkingdoms^35^ as a predictor variable, it explained an additional 13.6% of the variation in PD_es_ that was not explained by environmental predictors for angiosperms, 6.6% for palms and 13% for ferns (family-level; Supplementary Fig. S1). Models for PD_es_ and MPD_es_ were mostly consistent. We therefore present results on PD_es_ and only report major differences for MPD_es_ (see Supplementary Tables S4 and S5 and Supplementary Fig. S2 for MPD_es_).

### Dispersal filtering

Two dispersal-related variables, mainland species pool size (measured as species richness on the nearest mainland, MLSR^1^) and island isolation (measured as proportion of surrounding landmass times -1, SLMP^21^) were important for explaining angiosperm and palm PD_es_ (Table 1; Fig. 4); the third (geologic history) was rather unimportant. Mainland species pool size had the expected positive effect on PD_es_ of angiosperms and palms, but a negative effect on fern PD_es_ (Fig. 4). Island isolation showed the expected negative effect on palm PD_es_ (Fig. 4b; Supplementary Table S3), but no effect for ferns (Fig. 4a,b), and an effect opposite to our expectation for angiosperms, i.e. increasing PD_es_ with increasing isolation (Fig. 4a). Geologic history had no significant effects on PD_es_ (Fig. 4; Supplementary Table S3). However, for angiosperms and palms, geologic history had significant effects on MPD_es_, with highest values on continental shelf islands (Supplementary Table S5; Supplementary Fig. S2).

### Environmental filtering

Climatic variables were strong predictors for angiosperm PD_es_ – the only unimportant climatic variables were precipitation seasonality and climate change velocity in temperature since the last glacial maximum (CCVT; Table 1). Angiosperm PD_es_ showed a U-shaped relationship with annual mean temperature (increasing from ca. 10 °C onwards) and a hump-shaped relationship with annual precipitation (increasing up to ca. 3000 mm; Fig. 4a). For palm PD_es_, present-day seasonality in both temperature and precipitation were the most important climatic variables (Table 1). Palm PD_es_ showed a U-shaped relationship with temperature seasonality and a negative relationship with precipitation seasonality (Fig. 4b). For ferns, temperature and elevation range were most important for PD_es_ at the genus level, whereas precipitation, elevation range and CCVT were most important at the family level (Table 1). At both family and genus levels, fern PD_es_ increased with precipitation (Fig. 4).

### ***In-situ*** speciation

The most important variables related to speciation processes were island isolation (SLMP) and area for angiosperms and palms, and elevation range for ferns (Table 1). Whereas PD_es_ of angiosperms and palms showed the expected negative relationship with area, fern PD_es_ increased with area (Fig. 4). In contrast to our expectations and results for PD_es_, angiosperm MPD_es_ increased with area (Supplementary Fig. S2). For palms, PD_es_ decreased with increasing area only for areas larger than 100 km^2^ (Fig. 4b). Ferns were the only group for which elevation range showed the expected negative relationship with PD_es_ (Fig. 4; Table 1). However, we also found a decrease with increasing elevation range for angiosperm MPD_es_ (Supplementary Fig. S2). Island age did not have a significant effect on PD_es_ for any group when simultaneously accounting for other environmental variables (Supplementary Fig. S3).

### Sensitivity analyses

The results were robust to the choice of the species pool. Phylogenetic community metrics for all taxonomic groups based on the global island species pool were highly correlated to the corresponding metrics based on three different regional species pool delineations (all r > 0.86, p < 0.001; Supplementary Table S7, Supplementary Fig. S9). Relationships of phylogenetic structure with environmental predictors were almost the same across all four species pool delineations (Supplementary Fig. S10), and the variation explained remained considerably smaller for ferns than for angiosperms and palms when applying regional species pools (Supplementary Table S8).

## Discussion

Our results indicate that a large amount of variation in phylogenetic structure of island floras can be explained by physical and bioclimatic island characteristics. This was especially the case for angiosperms overall and palms, which had floras that were mostly clustered and with phylogenetic structure strongly related to island characteristics representing dispersal, environmental filtering and *in-situ* speciation processes. The considerable variation among the three plant groups suggests that drivers of phylogenetic structure have strong group-specific components, supporting hypothesis H1. In particular, environmental predictors explained more variation in phylogenetic structure of global island floras for angiosperms (49%) and palms (42%) than for ferns (18%), especially island characteristics preventing colonization and promoting speciation on islands (Table 1). Moreover, the strength and form of the relationships between phylogenetic structure and environmental variables showed different or even contrasting trends among the three groups, suggesting that dispersal filtering (H2), environmental filtering (H3) and *in-situ* speciation (H4) have differing effects on the phylogenetic structure of major plant groups on islands.

Dispersal-related environmental variables showed strikingly different patterns among our three study groups, with effects on phylogenetic structure being most pronounced for angiosperm and palm assemblages (Table 1). We found some support for hypothesis H2 in palms, which showed the expected negative effect of island isolation on PD_es_. The absence of many palm lineages on remote islands might be due to their comparatively large seeds and low dispersal ability, leading to phylogenetically impoverished palm floras (SLMP in Fig. 4b) and strong phylogenetic clustering on isolated islands (Supplementary Fig. S2). These results are consistent with previous findings^9^ which indicate that dispersal filters allow only few palm lineages to colonize and subsequently radiate *in-situ* on isolated islands (Supplementary Text S1). The higher values of angiosperm and palm MPD_es_ on continental shelf islands compared to continental fragments and oceanic islands (Supplementary Table S5) are also in line with these findings^9^ (see also ref. 10).

Contrary to our expectation of a decrease in phylogenetic overdispersion with increasing isolation (H2; Fig. 1), angiosperm PD_es_ was positively related to island isolation (SLMP; Fig. 4a). Phylogenetic composition of the most remote insular angiosperm floras indicates that immigrants came from multiple biogeographic source regions with distinct evolutionary histories, so that those floras cannot be attributed to a single mainland source pool (compare Box 4 in ref. 11). For instance, the Hawaiian angiosperm flora is composed of elements from all circum-Pacific regions^36^. Dispersal to remote islands is characterized by a strong non-deterministic component, i.e. rare stochastic dispersal events strongly influence the composition of isolated island floras^37^. Furthermore, the variety and multiple parallel evolutionary origins of long-distance dispersal modes in angiosperms^3^,^36^ make representatives from many angiosperm lineages capable of reaching remote islands (see Supplementary Methods S1 for a discussion about our assumption of phylogenetic signal in relevant traits). We therefore suggest that for angiosperms, the negative effect of dispersal filtering on phylogenetic structure is overshadowed by the positive effect of a wide variety of well-dispersing, distantly related clades.

For the phylogenetic structure of ferns, island isolation and geologic history were unimportant (Table 1), leading us to reject H2 for ferns. We attribute this result to small spores and the high dispersal ability of ferns (Supplementary Text S1). This is in accordance with recent findings indicating that fern diversity decreases less strongly with isolation than seed plant diversity, resulting in an overrepresentation of ferns on remote islands^38^,^39^, and that immigration is the main driver behind the assembly of island fern floras^40^. Accordingly, the size of the mainland species pool was positively related to PD_es_ for angiosperms and palms, as expected, but negatively for ferns (MLSR in Fig. 4). However, our mainland species pool size does not reflect phylogenetic composition of the species source pools, hampering direct inference on its effect on the assemblage structure of island floras.

Climatic variables were important predictors of phylogenetic structure across all groups (Table 1). This suggests an overall important role of environmental filtering for the phylogenetic structure of island floras, supporting H3. However, we found different environmental filters for the compared groups, supporting the idea that phylogenetically conserved physiological constraints of each group (Supplementary Text S1) determine which factors act as environmental filters^3^,^25^.

The increase of angiosperm PD_es_ above 10 °C is in line with recent findings for North American trees^26^ and the tropical niche conservatism hypothesis, which suggests that few angiosperm lineages are adapted to cold conditions due to tropical ancestry^25^. In contrast, the increase of PD_es_ with decreasing temperature below 10 °C is likely the result of small assemblages on cold, high latitude islands being made up of species from a few distantly related lineages that have independently evolved to tolerate harsh polar conditions (e.g. five species from five families on McDonald Island). Adaptations to drought also appear to be limited, as indicated by a strong positive relationship between angiosperm PD_es_ and annual precipitation up to 3000 mm annually (Fig. 4). The positive relationship of angiosperm PD_es_ with temperature seasonality is unexpected (not significant for MPD_es_), but may simply be the result of collinearity between annual means of temperature and seasonality (Supplementary Table S6).

For palms, an iconic tropical angiosperm family, it is intuitive that seasonality in temperature and precipitation were the most important climatic factors, negatively affecting PD_es_ (Table 1). This is consistent with the lack of adaptations to frost and drought in most palms^41^,^42^,^43^ (Supplementary Text S1). The positive relationship of PD_es_ with precipitation for ferns (Fig. 4) indicates that drought is also an environmental filter for ferns. This is consistent with most fern lineages being restricted to humid climates (Supplementary Text S1). Globally, fern diversity declines strongly along aridity and coldness gradients^38^, suggesting that ferns show strong climatic niche conservatism. We did not find any indication of Late Quaternary climate change filtering of insular phylogenetic diversity. The lack of a negative relationship with phylogenetic overdispersion across all groups is in line with recent findings for palm species richness on islands^42^ and suggests that extinctions due to past climate change affected species rather randomly across the phylogenetic tree or affected the global island species pool in a non-random way. Alternatively, islands might have been better buffered against past climate change than mainland areas^9^,^44^.

Supporting hypothesis H4, the strong negative effect of island area on angiosperm PD_es_ suggests an important role of *in-situ* speciation on large islands^8^. Interestingly, in contrast to PD_es_, angiosperm MPD_es_ was positively related to island area. We suggest that this difference stems from how the two metrics are calculated. For MPD_es_, classic island radiations involving many speciation events within a single lineage lead to phylogenetic clustering, while the same number of speciation events in several distant lineages leads to overdispersion. In contrast, both scenarios have the same effect on PD_es_, as each *in-situ* speciation event adds an additional branch to PD, regardless of its location in the tree. We therefore suggest that the negative effect of island area on PD_es_ indicates an increase in *in-situ* diversification with island area regardless of how closely related these island-endemic speciation events are. In contrast, the positive effect of island area on MPD_es_ indicates that larger islands tend to harbour more island-endemic radiations that are relatively species-poor and not closely related with each other. The largest islands are of continental origin and have had large floras from their formation onwards, possibly leaving less opportunity for large recent radiations.

In contrast, the largest palm radiations are on large continental islands (Supplementary Text S1). Accordingly, island area has a strong negative effect on both palm PD_es_ and MPD_es_, supporting *in-situ* radiations as a major driver of island palm diversity^9^ and highlighting the role of *in-situ* speciation for the assembly of biota^11^. This is in line with the negative effect of island isolation on palm PD_es_ and higher palm MPD_es_ on continental shelf islands (Fig. 4 and Supplementary Fig. S1), and the expectation of low dispersal ability and geographically-restricted gene flow in many palms (Supplementary Text S1).

Island area had only a minor effect on fern PD_es_ (Table 1). This can be explained by high levels of gene flow in ferns hampering *in-situ* speciation, even within large islands^29^. Even though there is considerable island endemism in ferns, this is usually lower than for angiosperms^36^ and mostly evolved via anagenesis^45^ (but see ref. 40). The only support for H4 for ferns is the negative relationship between phylogenetic overdispersion and elevation range, which might result from elevated diversification rates of ferns in tropical mountains^46^ (compare ref. 47 for parrots).

Our results do not support the expected effects of geologic history and oceanic island age on phylogenetic structure (Fig. 4 and Supplementary Fig. S3). Most oceanic islands are likely too young (mean age of 6.9 Ma) compared to the ages of the considered plant groups to show any apparent relationship with phylogenetic structure. Furthermore, there are examples of relict endemics on (young) volcanic archipelagos^48^ and radiations on (old) continental fragments^49^, suggesting a real lack of the proposed effects.

In addition to dispersal, environmental filtering and speciation, other factors may influence the phylogenetic structure of species assemblages, e.g. species interactions^2^,^3^,^11^. Most likely, however, species interactions should have greatest impact on community assembly at the local scale^3^,^4^. At large spatial scale, we found a strong imprint of regional biogeographic history on the phylogenetic patterns of island floras, with floristic subkingdoms accounting for a substantial proportion of variation in phylogenetic structure after considering environmental predictors (e.g. r^2^ = 0.33 for angiosperm MPD_es_; Supplementary Fig. S1). Exceptionally high PD_es_ in the Indo-Malaysian and Neocaledonian regions for example, can be explained by high numbers of ancient and relict endemic lineages^49^. Interestingly, the Neocaledonian region showed the highest PD_es_ and at the same time lowest MPD_es_ for angiosperms, indicating an imprint of species-rich radiations in addition to the relict lineages (Supplementary Fig. S1).

Using large regional species pools for the null models of PD_es_ and MPD_es_ instead of a global island species pool did not qualitatively change our results (Supplementary Fig. S10). Maybe, smaller regional species pools would allow a more insightful look into how the phylogenetic structure of island assemblages varies within regions^9^,^50^. However, the large regional and global species pools applied here allow comparing values of phylogenetic structure across regions, and are therefore appropriate for investigating global trends given the great dispersal ability of some members of the considered plant groups^51^,^52^ (Supplementary Text S1; see methods). The unexpected positive effect of island isolation on angiosperm PD_es_, for example, suggests that phylogenetic structure of the most remote islands is driven by colonization from multiple source pools (compare ref. 4).

Some environmental factors considered here as predictors of phylogenetic structure influence more than one of the examined processes. For example, island isolation and geology are important for both dispersal filtering and *in-situ* speciation, and elevation range can likewise affect both *in-situ* speciation and environmental filtering (Fig. 1). In addition to our hypothesized relationships, island area may also affect dispersal filtering via target area effects^53^ and contemporary and historical climate may affect speciation^9^. It is therefore intrinsically difficult to fully disentangle the relative importance of these processes for the structure of island assemblages. *In-situ* speciation and dispersal filtering in particular go hand in hand, as they should both increase in importance with island isolation. For a more rigorous assessment of the relative importance of speciation versus dispersal, information on the key species traits related to assembly processes would be needed. These data are currently not available at this large spatial and taxonomic scale of analysis, but assembling them is a major goal of ongoing ecological research^54^. Future research should thus incorporate information on dispersal traits (e.g. for well-studied angiosperm families), on actual *in-situ* speciation events and on species distributions in relation to environmental conditions, in order to disentangle the relative importance of different assembly processes. Meanwhile, our approach of comparing the relationships between phylogenetic structure and environmental variables among taxonomic groups with different predominant characteristics allows new inferences about how assembly processes act along large-scale environmental gradients and across species-rich clades.

In conclusion, our results suggest that the processes of dispersal filtering, environmental filtering and *in-situ* speciation leave strong signals in the phylogenetic structure of island assemblages. Phylogenetic structure of the three major plant groups studied here shows markedly different relationships with physical and bioclimatic variables that are linked to the three focal processes (Fig. 1). These differences can be attributed to contrasting traits among the plant groups, different adaptations to dispersal (e.g. predominantly animal-dispersed palms vs. wind-dispersed ferns) and environmental conditions (e.g. less drought-adapted ferns), and different levels of phylogenetic signal in relevant traits. The two metrics of phylogenetic structure we used, PD_es_ and MPD_es_, showed similar patterns in relation to dispersal and environmental filtering, but in the case of *in-situ* speciation they provide different perspectives, allowing us to separate the effects of radiations in single lineages and speciation in multiple lineages. Together, these findings provide new insights into how insular plant diversity originated, and underline the importance of dispersal, environmental filtering and speciation for generating global plant diversity patterns.

## Methods

### Floras and phylogenies

**Figure 2:**
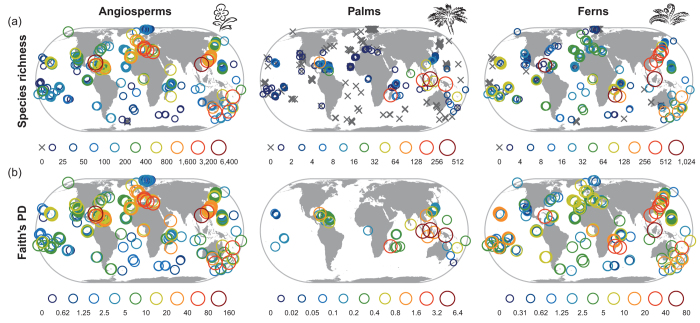
Global patterns of (a) species richness and (b) Faith’s phylogenetic diversity (PD) for all angiosperms, palms and ferns on islands. PD was calculated as the sum of all branch lengths representing the species of an island in a clade’s phylogeny^18^ excluding the root, based on a dated family-level phylogeny for angiosperms and on dated genus-level phylogenies for palms and ferns. PD is shown only for islands with at least two species of the focal group (363 of 375 islands for all angiosperms, 71 of 386 for palms and 234 of 328 for ferns). Species richness is given in numbers of species, PD in billion years. Numbers in legends indicate category borders. Maps were created using the statistical programming language R.

**Figure 3:**
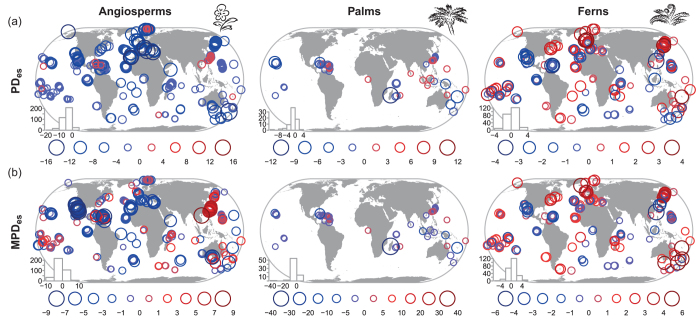
Phylogenetic structure of island floras. Phylogenetic structure is illustrated as deviations of (**a**) phylogenetic diversity (PD) and (**b**) mean pairwise phylogenetic distance (MPD) from null expectations based on insular species richness and a global species pool. Maps show results based on a dated family-level phylogeny for angiosperms and dated genus-level phylogenies for palms and ferns. The standardized effect sizes of PD and MPD (PD_es_ and MPD_es_) were based on null models randomly shuffling all included species at the tips of the trees. Negative values indicate phylogenetic clustering, positive values overdispersion. Only islands with at least two species of the focal groups are included (363 islands for angiosperms, 71 islands for palms and 234 islands for ferns). Embedded histograms give the frequency distributions of the mapped metrics. Numbers in legends indicate category borders. Maps were created using the statistical programming language R.

**Figure 4:**
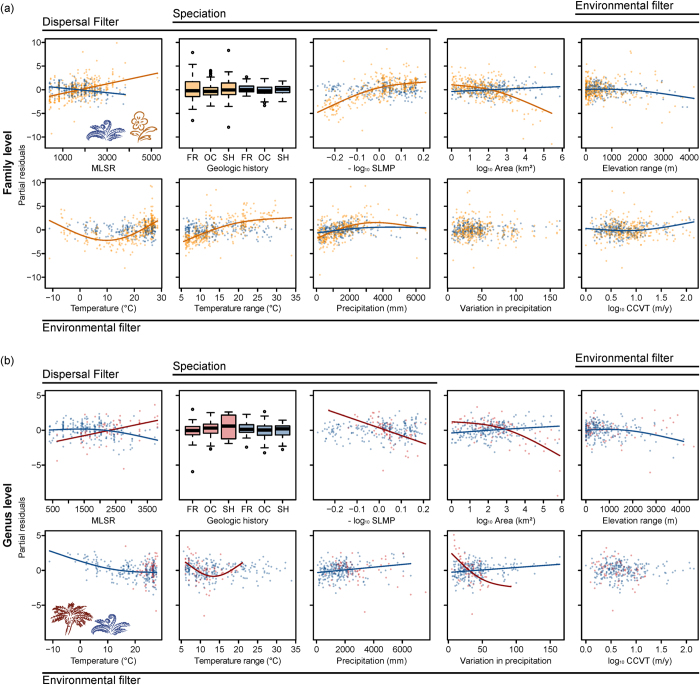
Environmental predictors of phylogenetic structure in island floras. Partial residual plots from averaged Generalized Additive Models illustrate the standardized effect size of phylogenetic diversity (PD_es_) of angiosperms, palms and ferns as a function of environmental predictors. Models included spatial eigenvectors to account for spatial autocorrelation. Regression lines are shown if the variable was significant in the averaged model. In (**a**), PD_es_ was based on dated family-level phylogenies of angiosperms (orange) and ferns (blue). In (**b**), PD_es_ was based on dated genus-level phylogenies of palms (red) and ferns (blue). Only islands with at least two species of the focal group are shown (363 islands for all angiosperms, 71 for palms and 234 for ferns). MLSR = Mainland species richness, SLMP = surrounding landmass proportion, CCVT = Late Quaternary climate change velocity; Geologic island types: FR = continental fragment, OC = oceanic island, SH = continental shelf islands.

Plant species lists for 393 marine islands were assembled from published floras, checklists and online databases (Figs 2 and 3), including 375 species lists with 32,446 species for all angiosperms (flowering plants), 386 lists with 1,143 species for palms and 328 lists with 3,689 species for ferns (Supplementary References S1). A comprehensive taxonomic standardization including family assignment was based on The Plant List (www.theplantlist.org) and other resources (Supplementary Methods S1).

We compared ferns with all angiosperms at the family level and with palms at the genus level, using the best available phylogenies (Supplementary Methods S1). For angiosperms, we used the dated phylogeny from ref. 31, which includes 560 angiosperm species from 335 families (Supplementary Fig. S4). We repeated analyses with the angiosperm phylogeny from ref. 32, a dated supertree including 379 families. Phylogenetic community metrics based on the two phylogenies were strongly correlated (all r > 0.98, p < 0.001; Supplementary Table S2), and therefore we only show results for the phylogeny from ref. 31. For palms, we used a complete and dated genus-level supertree^34^ (Supplementary Fig. S5). Our dated fern phylogeny^34^ was augmented by additional data, and includes 1,118 species representing most extant fern genera (Supplementary Methods S1; Supplementary Fig. S6).

To compare angiosperms and ferns, we pruned the phylogenies to family level (for details see Supplementary Methods S1) and then added all species from the island checklists to the family-level phylogenies as polytomies at 1/3 of the family stem node ages. To compare palms and ferns, phylogenies were pruned to genus level and species added as polytomies at 2/3 of the genus stem node ages^9^. We chose 1/3 for family-level phylogenies and 2/3 for genus-level phylogenies to account for the greater difference in stem node ages between species and families as compared to species and genera. In any case, comprehensive sensitivity analyses with the palm phylogeny have shown that the choice of age thresholds for polytomies does not qualitatively affect measures of phylogenetic assemblage structure^9^ because metrics are predominantly influenced by long branches in older parts of phylogenies (see Supplementary Methods S1 for discussion about the resolution of the phylogenies).

### Phylogenetic community metrics

Our first measure of phylogenetic structure was the standardized effect size (PD_es_) of phylogenetic diversity (PD). To obtain PD_es_, we first calculated Faith’s PD for each island and taxonomic group as the summed length of unique branches leading to species from that island in the phylogeny of that taxonomic group, excluding the root^18^. Because PD is strongly correlated with species richness^7^,^27^ (Supplementary Table S1), we then calculated the deviation of PD from a global null expectation (PD_0_), which was calculated as the mean value of PD over 1,000 trees generated by randomly reshuffling the species at the tree tips. Since the variance in deviation from null expectations is related to species richness, the deviation from the null expectation was divided by the standard deviation of the PD values from the reshuffled trees (PD_0sd_) to obtain PD_es_ (Equation 1).


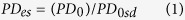


As a second measure of phylogenetic structure, we calculated the standardized effect size of mean pairwise phylogenetic distance (MPD_es_) of species on an island as the deviation of the real mean pairwise phylogenetic distance (MPD) for that island from the null model mean (MPD_0_, calculated like PD_0_) divided by the null model standard deviation (MPD_0sd_; Equation 2).


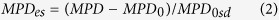


MPD_es_ multiplied by –1 equals the commonly used net relatedness index^6^. We did not multiply by –1, to keep MPD_es_ values comparable to PD_es_. Hence, negative values of PD_es_ and MPD_es_ indicate phylogenetic clustering whereas positive values indicate overdispersion, i.e. higher values of PD and MPD than expected by chance. Phylogenetic community metrics could only be calculated for communities of at least two species. This reduced our dataset to 363 islands for angiosperms, 71 for palms and 234 for ferns.

Because we were interested in global trends rather than within-region variation, we used the global species pool of all island species in the null models of phylogenetic community metrics for the main analyses (compare ref. 9). Identifying a mainland species pool for each island would be the ideal way to test dispersal and environmental filtering of the initial colonizing lineages and subsequent *in-situ* speciation, but (i) knowledge on plant species distributions on the mainland is limited and (ii) there might not even be a single (discrete) mainland pool for most islands. In contrast to the simplified situation in the equilibrium theory of island biogeography where a target island recruits colonists from one closest mainland source pool, there is mounting evidence that floras of remote oceanic islands recruit colonists from multiple distant biogeographic regions including other large islands^36^,^55^. The sister species of the Hawaiian endemic *Acacia koa*, for example, was recently discovered to be the La Réunion endemic *Acacia heterophylla*^51^ (18,000 km apart). Hence, dispersal filtering acts across spatial scales^3^ and it would be misleading to consider only a single regional source pool per island. To test for environmental filtering due to missing adaptations to certain macroclimatic conditions, it is also important to include species that are not adapted into the species pool in the first place. We therefore used the global species pool of island species to identify how island assemblages are structured with regard to all successful colonizers, i.e. species that have made it to any island, and all species evolved on islands. To account for the multiple spatial scales of dispersal, we performed sensitivity analyses using phylogenetic community metrics based on three different, large regional species pool delineations. Regional species pools included all species of all islands that belong to a particular floristic realm modified after Takhtajan^35^ (Holarctis, Neotropis, Palaeotropis, Holantarctis including Australis), that belong to a major ocean basin (Pacific, Atlantic, Indian Ocean), or that are located within 10,000 km around each target island^56^ (Supplementary Fig. S9).

### Environmental predictors

To test for dispersal filtering on islands (Fig. 1), we analysed the relationships between phylogenetic structure and island isolation, geologic history, and the size of the mainland species pool. For a measure of island isolation that accounts for stepping stone islands and the amount of source landmass, we calculated the proportion of landmass surrounding each island in distance classes up to 5,000 km radius (SLMP) following ref. 21, multiplied by –1. We assembled information on island geologic history from encyclopaedias, distinguishing between continental shelf islands (with a potential mainland connection during the last glacial maximum), continental fragments (separated from continents by tectonic movements) and oceanic islands (never connected to mainland). Since species with distributions restricted to mainlands were not included in our species pools, we used the species richness of the nearest mainland grid cell (variable MLSR) from a co-kriging model of vascular plant species richness^1^, to account for the size of the mainland species pool available for island colonization.

We used past and present climate as well as environmental heterogeneity of islands to test for the effect of environmental filtering on phylogenetic structure. As measures of present climate, we considered mean annual temperature (°C), annual precipitation (mm), temperature seasonality (annual range; °C) and precipitation seasonality (coefficient of variation of monthly means; Table 1). To test for paleoclimatic effects, we used Late Quaternary climate change velocity of temperature (CCVT)^57^. This measure represents the speed (m y^−1^) required to keep up with changing climate since the last glacial maximum (21000 y BP), considering topographic heterogeneity. In addition, we used elevation range (m) as a measure of environmental heterogeneity. Environmental variables were taken from ref. 17.

We considered island isolation, geologic history, environmental heterogeneity and area as factors influencing *in-situ* speciation on islands because of their effects on gene flow (Fig. 1). Island area (km^2^) was taken from ref. 17. In addition, we used island ages (My), gathered for 202 volcanic and uplifted seafloor islands from literature, as a proxy for time available for speciation.

### Statistical analyses

We used Generalized Additive Models (GAM), which are a powerful tool to detect non-linear relationships, to examine the relationships between phylogenetic metrics (PD_es_, MPD_es_) and environmental predictors. The modelling was performed separately for the global species pool metrics and as sensitivity analysis for metrics based on the three different regional species pool delineations (Supplementary Fig. S9). If not stated otherwise, results are shown and discussed based on the global island species pool. Area, SLMP and CCVT were log_10_-transformed due to strongly skewed frequency distributions. All predictors except geologic history (which was categorical) were added to models as penalized regression splines with up to two degrees of freedom. We used Akaike’s Information Criterion corrected for small sampling sizes (AIC_c_) to select best models from all possible candidate models and performed model averaging^58^. We tested for spatial autocorrelation in response variables and in residuals of the best models by comparing global Moran’s *I* values for varying neighbourhood structures considering k = 1–25 nearest neighbours (Supplementary Figs S7 and S8). To account for spatial autocorrelation in model residuals, we applied spatial eigenvector filtering^59^ (see Supplementary Methods S2 for details). Model selection and model averaging were performed including the set of spatial filters identified for the best non-spatial models, and the new best spatial models as well as averaged models were used for representation of results. We report pseudo R^2^-values derived from linear models of observed vs. predicted values from the GAMs, disregarding the spatial filters in the predictions, to estimate variation explained by environmental predictors alone. We used cumulative AIC_c_ weights from all candidate models including a given variable as a measure of variable importance^58^.

To account for effects of regional biogeographic history on present-day phylogenetic patterns, we reran the model and spatial filter selection procedure including floristic subkingdom membership^35^ as an additional predictor and performed model averaging. To assess the influence of island age on the phylogenetic structure of island floras, we used the subset of oceanic islands for which age of emergence was available (*n* = 187 islands for angiosperms, 31 for palms, 138 for ferns). We used the same model and spatial filter selection procedure as for the full dataset. Geologic history was not included in these models as these islands were all of oceanic origin.

Phylogenetic community metrics and environmental predictor variables are available in Supplementary Dataset S1.

## Additional Information

**How to cite this article**: Weigelt, P. *et al.* Global patterns and drivers of phylogenetic structure in island floras. *Sci. Rep.* **5**, 12213; doi: 10.1038/srep12213 (2015).

## Supplementary Material

Supplementary Information

Supplementary Dataset 1

## Figures and Tables

**Table 1:** Variable importance estimated from Generalized Additive Models for the standardized effect size of phylogenetic diversity (PD_es_) of angiosperms, palms and ferns on islands.

**Variable**	**Process (**Fig. 1)			**Family-level phylogeny**		**Genus-level phylogeny**	
	**Disp**	**Spec**	**Env**	**Angiosperms**	**Ferns**	**Palms**	**Ferns**
Mainland species richness (MLSR)	X			**1**	**1**	**0.94**	**1**
Geologic history (fragment, shelf, oceanic)	X	X		0.46	0.35	0.23	0.22
Surrounding landmass proportion (SLMP; –1 x log_10_)	X	X		**1**	0.27	**0.94**	0.35
Island area (km^2^; log_10_)		X		**1**	0.75	**0.97**	0.78
Elevation range (m)		X	X	0.54	**0.96**	0.27	**0.93**
Annual mean temperature (°C)			X	**1**	0.31	0.31	**1**
Temperature seasonality (annual range; °C)			X	**1**	0.27	**0.88**	0.27
Annual precipitation (mm)			X	**1**	**0.98**	0.47	**0.88**
Precipitation seasonality (coefficient of variation)			X	0.34	0.43	**0.98**	**0.84**
Late Quaternary climate change velocity in temperature (m y^–1^; CCVT; log_10_)			X	0.37	**0.98**	0.45	0.27

Importance was assessed as the sum of AIC_c_ (Akaike’s Information Criterion corrected for small sampling sizes) weights of all models including the focal variable. Apart from all possible combinations of the predictor variables, all candidate models included spatial eigenvectors to account for spatial autocorrelation. Angiosperm PD_es_ was calculated based on a dated family-level phylogeny, palm PD_es_ based on a dated genus-level phylogeny. Fern PD_es_ was based on dated phylogenies at both family and genus levels. Islands with at least two species of the focal group were considered (363 islands for angiosperms, 71 for palms, 234 for ferns). Columns Disp (dispersal filtering), Spec (*in-situ* speciation) and Env (environmental filtering) indicate to which hypothesized process influencing PD_es_ the variables relate. Values larger than 0.8 are printed in bold.
