# Supplementary material for: Global patterns and drivers of phylogenetic structure in island floras
Source: Sci Rep. 2015 Jul 22;5:12213. doi: 10.1038/srep12213 (PMC4510489; doi:10.1038/srep12213)
Supplement: Supplementary Information [file srep12213-s1.pdf]

Supplementary information

Weigelt, P., Kissling, W.D., Kisel, Y., Fritz, S.A., Karger, D.N., Kessler, M., Lehtonen, S., Svenning, J.-C. and Kreft, H. Global patterns and drivers of phylogenetic structure in island floras. *Scientific Reports*.

Content

Supplementary Text S1. Ecological and biogeographical characteristics of angiosperms, palms and ferns relevant to the hypothesized relationships between environmental factors and phylogenetic diversity and structure.

Supplementary Methods S1. Floras and phylogenies.

Supplementary Methods S2. Statistical models and spatial autocorrelation.

Supplementary Table S1. Pearson correlations of phylogenetic community metrics within angiosperms, palms and ferns on islands worldwide.

Supplementary Table S2. Pearson correlations of phylogenetic community metrics among angiosperms, palms and ferns on islands worldwide.

Supplementary Table S3. Best Generalized Additive Models based on Akaike's Information Criterion corrected for small sampling sizes of the relationships of the standardized effect size of phylogenetic diversity of angiosperms, palms and ferns with environmental factors on islands.

Supplementary Table S4. Variable importance estimated from all possible multi-predictor Generalized Additive Models for the standardized effect size of mean pairwise phylogenetic distance of angiosperms, palms and ferns on islands in dependence on environmental predictors.

Supplementary Table S5. Best Generalized Additive Models based on Akaike's Information Criterion corrected for small sampling sizes of the relationships of the standardized effect size of mean pairwise phylogenetic distance of angiosperms, palms and ferns with environmental factors on islands.

Supplementary Table S6. Pearson correlations among predictor variables used to explain phylogenetic structure of island floras.

Supplementary Table S7. Pearson correlations among phylogenetic community metrics calculated with a global island species pool and three different regional species pool delineations.

Supplementary Table S8. Variation explained by Generalized Additive Models of the standardized effect size of phylogenetic diversity and mean pairwise phylogenetic distance of angiosperms overall, palms and ferns on islands based on a global island species pool and three different regional species pool delineations as response variables and environmental predictor variables.

Supplementary Figure S1. Partial residual plots for Takhtajan's floristic subkingdoms derived from averaged Generalized Additive Models of the relationships of the standardized effect sizes of phylogenetic diversity and mean pairwise phylogenetic distance of angiosperms, palms and ferns with environmental factors on islands.

Supplementary Figure S2. Partial residual plots from averaged Generalized Additive Models of the relationships of the standardized effect size of mean pairwise phylogenetic distance of angiosperms, palms and ferns with environmental factors on islands.

Supplementary Figure S3. Partial residual plots for island geologic age derived from averaged Generalized Additive Models of the relationships of the standardized effect sizes of phylogenetic diversity and mean pairwise phylogenetic distance of angiosperms, palms and ferns with environmental factors on islands.

Supplementary Figure S4. Dated angiosperm phylogeny used to analyse the phylogenetic structure of island floras.

Supplementary Figure S5. Dated palm phylogeny used to analyse the phylogenetic structure of island floras.

Supplementary Figure S6. Dated fern phylogeny used to analyse the phylogenetic structure of island floras.

Supplementary Figure S7. Moran's *I* correlograms of spatial autocorrelation for the standardized effect size of phylogenetic diversity of angiosperms, palms and ferns on islands.

Supplementary Figure S8. Moran's *I* correlograms of spatial autocorrelation for the standardized effect size of mean pairwise phylogenetic distance of angiosperms, palms and ferns on islands.

Supplementary Figure S9. Three different delineations of regional species pools used in sensitivity analyses.

Supplementary Figure S10. Species pool sensitivity analyses for environmental predictors of phylogenetic structure in island floras.

Supplementary References S1. Literature used to compile the global dataset of angiosperm, palm and fern species composition on 393 islands worldwide.

Supplementary References S2. References cited in this supplement

Supplementary Text S1. Ecological and biogeographical characteristics of angiosperms, palms and ferns relevant to the hypothesized relationships between environmental factors and phylogenetic diversity and structure (Fig. 1).

Angiosperms overall. Dispersal modes in angiosperms range widely in their chance of long distance dispersal, from wind and ocean dispersal to underground fruits depositing seeds next to parent plants. Some angiosperm families show remarkably little variation in dispersal modes[1] and seed mass is often conserved within genera and families[2] and larger lineages[3]. Many basal lineages also show conserved physiological tolerances and habitat affinities[4,5], with half of all families being restricted to the tropics, mainly because they lack adaptations to tolerate coldness and frost[6]. Together, these features make angiosperms an ideal model group for investigating dispersal and environmental filtering. Angiosperms also include many spectacular examples of radiations on archipelagos (e.g. Hawaiian lobeliads[7]) or large single islands (e.g. *Dypsis* on Madagascar[8]), and of relict endemic island lineages, even at the taxonomic rank of families (e.g. Amborellaceae on New Caledonia[9]).

Palms. Many species of the angiosperm family of palms (Arecaceae) are characterized by larger fruits and seeds than most other angiosperm families[3]. Only a few species with floating fruits (e.g. *Cocos*, *Nypa*) or fruits eaten by large-bodied flying birds are capable of intermediate- to long-distance dispersal; most species are strongly dispersal limited[10]. For example, species with large fruits that are predominantly dispersed by non-volant mammals[10] rarely reach remote islands. Accordingly, many higher-level palm taxa are restricted to certain biogeographic regions or islands[11]. Palms are characterized by a strictly subtropical to tropical distribution with a strong temperature and precipitation-dependent diversity gradient increasing towards the inner tropics[12-14]. The vast majority of higher-level palm taxa cannot survive seasonality and cold because of their soft and water-rich tissue, inability to undergo dormancy and lack of frost tolerance, particularly of the apical meristems[11,12]. Palms include many island radiations (e.g. *Pritchardia* on Hawaii[7], *Dypsis* on Madagascar[8], *Coccothrinax* and *Copernicia* on Cuba[10]) and show evidence of cladogenesis even on small islands (e.g. *Howea* on Lord Howe Island[15]).

Ferns. Few fern clades include island radiations that reach the magnitude of angiosperms (but see *Cibotium* and *Diellia* on Hawaii[16]). The lower tendency of ferns to show high *in-situ* speciation rates is likely due to their high dispersal ability[16-18]. Ferns have small wind-dispersed spores, and most species produce bisexual gametophytes capable of self-fertilization[19]. Consequently, ferns tend to have lower diversification rates and larger ranges than angiosperms, and speciation through hybridization and polyploidization makes up a larger part of fern speciation events[17,19]. Hence, dispersal filtering and *in-situ* speciation should be of minor importance for phylogenetic diversity of island ferns. However, environmental filtering might play an important role in ferns due to their strong dependence on humidity which is known to have a particularly strong limiting influence on species richness in this group[20]. Very few fern lineages have evolved adaptations to drought, which may be explained by eco-

physiological constraints like their lack of active stomatal control, their need for water for sperm movement and their comparatively simple xylem anatomy[21,22]. Accordingly, fern floras on islands may be unbalanced compared to mainland floras, with an overrepresentation of families characterized by adaptations to islands' ecological niches[18].

Supplementary Methods S1. Floras and phylogenies.

We assembled plant species lists for marine islands from floras, checklists and online databases. Based on the more than 1000 species lists in our database, we only included lists which claimed completeness in our analysis (see Supplementary References S1). The final selection covered 393 islands, 375 for all angiosperms (flowering plants), 386 for palms and 328 for ferns. All species names (including subspecies name and author information if available) were matched to the working list of all known plant species, the Plant List, version 1.0 (www.theplantlist.org/1/). Genus names not found in the Plant List were manually checked for mistakes and validity according to Mabberley's Plant-Book[23]. Species names were matched to the Plant List using fuzzy matching and replaced by names accepted by the Plant List if they were found to be synonyms. If a name could not be matched or its taxonomic status in the Plant List was unresolved, we used the Taxonomic Name Resolution Service provided by iPlant (tnrs.iplantcollaborative.org, accessed May 2, 2013) for taxonomic match-up. Species names that were matched but not resolved by either service or that were not matched at all were used in their matched or original form, respectively (97.7% matched / 85.8% resolved for angiosperms, 97.2% matched / 65.3% resolved for ferns, 99.8% matched / resolved for palms; in total 95% using the Plant List, 5% using iPlant). All names entered further analyses at the species level. Family assignment followed the Plant List, which corresponds to the Angiosperm Phylogeny Group (APG) classification III[24]. To match the taxonomic concepts of the fern phylogeny, and to acknowledge recent advances in fern taxonomy, all fern names were additionally subjected to a comprehensive and careful taxonomic check (by M.K. and S.L.) so that genus and family assignments were up to date.

In order to link the species from the island checklists to the phylogenetic trees, phylogenies were pruned to family level (angiosperms and ferns) or genus level (palms and ferns) and species added as polytomies according to their family or genus membership.

For angiosperms, the original phylogeny[25] considered DNA sequence data for 560 species from 335 families and 45 orders, and was simultaneously estimated and dated using Bayesian methods based on 35 fossils and an additional age constraint for the root of the tree[25]. For comparison with ferns, we pruned the phylogeny to family level (Supplementary Fig. S4). Five pairs of families that would otherwise not be monophyletic were merged (Supplementary Fig. S4). 60 families representing 935 species were missing from the phylogeny and were manually added to the tree according to ref. 26, the Angiosperm Phylogeny Group (APG) classification III[24], and information on the Angiosperm Phylogeny Website[27]. In the cases of Achatocarpaceae, Alismataceae, Butomaceae, Cymodoceaceae, Juncaginaceae, Posidoniaceae, Potamogetonaceae and Scheuchzeriaceae the position in the tree and ages relative to adjacent clades could be adopted from ref. 26. In all other cases, age estimates were not available (Anisophylleaceae, Aphanopetalaceae, Apodanthaceae, Balanophoraceae, Bonnetiaceae, Brunelliaceae, Calceolariaceae, Campynemataceae, Centrolepidaceae, Clusiaceae , Connaraceae,

Corsiaceae, Cynomoriaceae, Cytinaceae, Dipentodontaceae, Dirachmaceae, Elatinaceae, Gisekiaceae, Gyrostemonaceae, Haemodoraceae, Hydatellaceae, Joinvilleaceae, Linderniaceae, Lophiocarpaceae, Loranthaceae, Mayacaceae, Mitrastemonaceae, Montiaceae, Nartheciaceae, Nitrariaceae, Nothofagaceae, Olacaceae, Pandaceae, Paracryphiaceae, Pennantiaceae, Pentaphragmataceae, Peraceae, Phrymaceae, Phyllanthaceae, Picramniaceae, Rhipogonaceae, Ruppiaceae, Salvadoraceae, Schlegeliaceae, Siparunaceae, Sphenocleaceae, Stegnospermataceae, Tetrachondraceae, Tovariaceae, Trimeniaceae, Triuridaceae, Xyridaceae). Families thought to be the sister clade of a family in the tree were added at 2/3 of the stem age of the family in the tree. Families thought to be sister to larger clades were added half way between nodes. For the calculation of phylogenetic community metrics, the 32,446 angiosperm species from the island checklists were added to the family-level phylogeny as polytomies at 1/3 of the family stem node ages. The final phylogeny pruned to include only species present in the considered island floras comprised 315 families and merged groups (Supplementary Fig. S4).

The palm phylogeny was based on a complete genus-level supertree of palms, dated using a Bayesian relaxed molecular clock approach with uncorrelated rates and calibrated using four palm fossil taxa and a stem node age constrained to 110 to 120 Ma[28]. For comparison with ferns, we pruned the phylogeny to genus level (Supplementary Fig. S5). For the calculation of phylogenetic community metrics, the 1143 palm species from the island checklists were added to the phylogeny as polytomies at 2/3 of the genus stem node ages[10]. All palm species included in the species checklists were represented by genera in the phylogeny. The final phylogeny pruned to only include species present in the considered island floras comprised 118 genera (Supplementary Fig. S5).

For ferns, we used a dated phylogeny based on a global fern phylogeny[29]. This dataset was updated by querying GenBank release 184 (June 15 2011), complemented with additional data not included in the queried release (KJ628500-KJ628963; KJ716370-KJ716414), and filtered to retain only those taxa that were represented in the dataset by at least two genes (one of which had to be *rbcL*) and more than 1000 base pairs of sequence data. Furthermore, the most similar taxa (defined by the pairwise distance of aligned sequences) were removed until no pair of taxa had pairwise distance less than 0.5%. This resulted in a taxonomically broad sample of 1118 taxa representing most extant fern genera. Molecular dating was based on uncorrelated exponential relaxed clock analysis in Beast 1.7.3[30], using 42 fossil calibrated nodes and a partially constrained starting tree produced in RAxML 7.3.0[31,32]. For comparison with angiosperms, we pruned the phylogeny to family level following the classification from ref. 33, and for comparison with palms, we pruned the phylogeny to genus level (Supplementary Fig. S6). A group of nine genera that would otherwise not be monophyletic was merged (Polypodiaceae A in Supplementary Fig. S6: *Lemmaphyllum*, *Lepidomicrosorium*, *Lepisorus*, *Leptochilus*, *Microsorum*, *Neocheiropteris*, *Neolepisorus*, *Paragramma* and *Tricholepidium*). The genus *Odontosoria* was split into an old world clade and a new world clade to avoid polyphyly. 25 missing genera representing 146 species were added to the tree manually according to information

from the literature (Supplementary Fig. S6). Genera thought to be located inside genera in the tree were merged with the already present genera. Genera thought to be the sister clade of a genus in the tree were added at 2/3 stem age of the genus in the tree (*Aenigmopteris*, *Austrogramme*, *Cerosora*, *Cheiroglossa*, *Oenotrichia*, *Paraselliguea*, *Scoliosorus*, *Syngramma*, *Taenitis* and *Vaginularia*). Genera thought to be sister to larger clades were added half way between nodes (*Ananthacorus* and *Trachypteris*). The genus *Adenoderris* (one species with one occurrence on Jamaica) was excluded due to its unknown phylogenetic position. For the calculation of phylogenetic community metrics, the 3689 fern species from the island checklists were added to the family-level phylogenies as tips at 1/3 of the family stem node ages and to the genus-level phylogenies as polytomies at 2/3 of the genus stem node ages. We chose 1/3 in the family phylogenies to account for the higher discrepancy between stem node ages of families and species when compared to genera and species in the genus-level phylogenies (2/3 stem node age). However, comprehensive sensitivity analyses of the palm phylogeny show that the specific age thresholds for polytomies do not qualitatively affect patterns and determinants of phylogenetic community structure[10] because the metrics are predominantly influenced by long branch lengths in the older parts of the phylogeny. The final phylogenies pruned to include only species present in the considered island floras comprised 42 families and 168 genera (Supplementary Fig. S6).

Angiosperms, palms and ferns differ in age, number of species and major clades, and number of islands inhabited. However, the fern phylogeny encompasses a similar time span to the angiosperm phylogeny and an intermediate number of species compared to angiosperms and palms (Supplementary Figs S4, S5 and S6). In contrast to common belief, extant fern diversity is not older than angiosperm diversity; the largest fern lineages diversified in response to diversification in angiosperms[34]. Differences between angiosperms, palms and ferns in the importance of dispersal, environmental filtering and diversification for phylogenetic assembly can therefore directly be compared and linked to differences in dispersal- and speciation-related traits.

The comparison between family- and genus-level analyses for ferns enabled us to scrutinize the sensitivity of our analyses towards the resolution of the phylogenies. Both levels, however, provide sufficient detail to address our hypotheses (Fig. 1) and to disentangle patterns and determinants of phylogenetic structure of island floras as most variation in branch lengths is in basal parts of phylogenies. Thus, higher resolution in relationships among species is not expected to considerably influence general patterns and dependencies (compare sensitivity analyses in ref. 10). In addition, dispersal-related traits and environmental adaptations are phylogenetically conserved in many large and old clades[2,3,6]. Thus filtering mechanisms should affect phylogenetic structure independent of whether family- or genus-level phylogenies have been used. Furthermore, usually young island radiations[35] are clearly distinguishable from relict lineages which often go back way beyond genus and even family level (e.g. Amborellaceae[9]) even in genus or family-level phylogenies with species appended as polytomies. In fact, there are plenty of examples of island radiations producing up to hundreds of closely related species[7,36], leading to clustered island assemblages.

Our hypotheses on dispersal and environmental filtering as drivers of phylogenetic patterns assume phylogenetic signals in traits which vary with phylogenetic scale[37]. The more of the tree of life is encompassed, the more conservative the traits should be[4]. However, if traits of clades of different biogeographic regions have converged, conservatism may diminish[4,37], hampering comparisons among phylogenies. In fact, we could not test for a phylogenetic signal of traits in the phylogenies, but previous studies suggest that dispersal related traits like seed size and dispersal mode, and adaptations to climates are phylogenetically conserved in many large and old clades[2,3,6,38] (Supplementary Text S1). Furthermore, our results may help to understand patterns arising from different levels of phylogenetic signal in traits. The environmental models explained varying proportions of variance for angiosperms, palms and ferns (Table 1) suggesting differences in both predominant traits and levels of trait conservatism.

Tree editing was performed with R statistical software version 3.0.1 (R development Core Team, available at cran.r-project.org) using the package *ape*[39]. Phylogenetic community metrics were calculated using the package *picante*[40].

Supplementary Methods S2. Statistical models and spatial autocorrelation.

To account for spatial autocorrelation in model residuals of the best non-spatial model of each plant group, we applied spatial eigenvector filtering[41]. We applied principal coordinate analysis to a neighbourhood matrix (PCNM) to deconstruct geographic distances between island centroids into orthogonal spatial eigenvectors. Spatial distances were truncated by exchanging distances larger than 1000 km by 4000 km to emphasize spatial autocorrelation at relatively small scales[41]. All eigenvectors with positive eigenvalues were considered as they represent positive spatial autocorrelation at different spatial scales. Following ref. 42, we consecutively added spatial filters as linear effects to the best models until residual spatial autocorrelation was no longer significant. In each round, the spatial filter that best reduced residual Moran's I values was retained in the model for the next round. Moran's I values were calculated for varying neighbourhood structures considering the $k = 1$ to 25 nearest neighbours; the highest significant Moran's I value was always considered. Afterwards, the model selection procedure to find the best model and model averaging based on Akaike's Information Criterion corrected for small sampling sizes were repeated, with the identified set of spatial eigenvectors included in each model.

Analyses were performed with R statistical software version 3.0.1 (R development Core Team, available at cran.r-project.org) using packages *mgcv*[43] for Generalized Additive Models, *MuMIn*[44] for model selection and averaging, *vegan*[45] for PCNM and *spdep*[46] for spatial autocorrelation assessment.

Supplementary Table S1. Pearson correlations of phylogenetic community metrics within angiosperms, palms and ferns on islands worldwide. Metrics were calculated for angiosperms based on the dated family-level phylogeny from ref. 25, and for palms based on a dated genus-level phylogeny. For comparison, metrics for ferns were calculated using phylogenies at both family and genus levels. MPD_{es} = standardized effect size of mean pairwise phylogenetic distance, PD_{es} = standardized effect size of phylogenetic diversity (PD). n = 363 islands for all angiosperms, n = 71 islands for palms and n = 234 islands for ferns. Coefficients and p-values were corrected for spatial autocorrelation following ref. 47. Significance: * p < 0.05, ** p < 0.01, *** p < 0.001.

Taxon	Metric	log_{10} SR	log_{10} PD	PD_{es}
**Angiosperms**	**log_{10} PD**	0.99 ***		
	**PD_{es}**	-0.39 ***	-0.29 **	
	**MPD_{es}**	0.04	0.10	0.56 **
**Ferns (family)**	**log_{10} PD**	0.96 ***		
	**PD_{es}**	0.03	0.23 ***	
	**MPD_{es}**	0.07	0.24 ***	0.86 ***
**Palms**	**log_{10} PD**	0.83 ***		
	**PD_{es}**	-0.36 **	0.04	
	**MPD_{es}**	-0.59 ***	-0.32 **	0.84 ***
**Ferns (genus)**	**log_{10} PD**	0.97 ***		
	**PD_{es}**	-0.03	0.17 *	
	**MPD_{es}**	0.05	0.22 ***	0.86 ***

Supplementary Table S2. Pearson correlations of phylogenetic community metrics among angiosperms, palms and ferns on islands worldwide. Metrics were calculated for angiosperms based on two dated family-level phylogenies[25,26], and for palms based on a dated genus-level phylogeny. For comparison, metrics for ferns were calculated using phylogenies at both family and genus levels. MPD_{es} = standardized effect size of mean pairwise phylogenetic distance, PD_{es} = standardized effect size of phylogenetic diversity (PD). n = 363 islands for all angiosperms, n = 71 islands for palms and n = 234 islands for ferns. Coefficients and p-values were corrected for spatial autocorrelation following ref. 47. Significance: * p < 0.05, ** p < 0.01, *** p < 0.001.

Metric	Taxon	Angiosperms (Davies *et al.*)	Angiosperms (Bell *et al.*)	Ferns (family)	Ferns (genus)
**PD**	**Angiosperms (Bell *et al.*)**	1.00 ***			
	**Ferns (family)**	0.71 ***	0.71 ***		
	**Ferns (genus)**	0.71 ***	0.70 ***	1.00 ***	
	**Palms**	0.83 ***	0.83 ***	0.78 ***	0.78 ***
**PD_{es}**	**Angiosperms (Bell *et al.*)**	0.98 ***			
	**Ferns (family)**	0.08	0.01		
	**Ferns (genus)**	-0.01	-0.07	0.91 ***	
	**Palms**	0.04	0.22	-0.27	-0.11
**MPD_{es}**	**Angiosperms (Bell *et al.*)**	0.99 ***			
	**Ferns (family)**	0.31 ***	0.30 **		
	**Ferns (genus)**	0.30 ***	0.29 **	0.99 ***	
	**Palms**	0.41 **	0.40 **	-0.28	-0.28

Supplementary Table S3. Best Generalized Additive Models based on Akaike's Information Criterion corrected for small sampling sizes of the relationships of the standardized effect size of phylogenetic diversity (PD_{es}) of angiosperms, palms and ferns with environmental factors on islands. In addition to the parameters shown here, the models included spatial eigenvectors to account for spatial autocorrelation. For angiosperms, PD_{es} was calculated based on a dated family-level phylogeny, and for palms based on a dated genus-level phylogeny. For comparison, PD_{es} of ferns was calculated using phylogenies at both family and genus levels (n = 363 islands for all angiosperms, n = 71 islands for palms and n = 234 islands for ferns). R^2 is a partial R^2 for the predictor variables, removing the effect of the spatial eigenvectors; edf = effective degrees of freedom; column t/F-value contains t-values in case of intercepts and linear effects (edf =1) and F-values in case of smooth terms (edf > 1); MLSR = Mainland species richness, SLMP = surrounding landmass proportion, CCVT = Late Quaternary climate change velocity.

Term	Estimate	Std. error	edf	t/F-value	p-value	R^2
**Angiosperms**						0.49
Intercept	-4.185	0.093	1.000	-45.003	< 0.001	
Elevation range (m)			1.318	3.064	0.061	
$\log_{10}$ Area (km²)			1.852	19.346	< 0.001	
MLSR			1.000	28.613	< 0.001	
Precipitation (mm)			1.926	30.367	< 0.001	
-1 x $\log_{10}$ SLMP			1.870	32.029	< 0.001	
Temperature (°C)			1.997	26.865	< 0.001	
Temperature range (°C)			1.855	28.384	< 0.001	
**Ferns (family)**						0.15
Intercept	0.253	0.072	1.000	3.518	0.001	
Elevation range (m)			1.751	4.600	0.012	
$\log_{10}$ Area (km²)			1.000	5.536	0.019	
$\log_{10}$ CCVT (m/y)			1.901	6.551	0.002	
MLSR			1.000	19.900	< 0.001	
Precipitation (mm)			1.775	8.257	< 0.001	
**Palms**						0.52
Intercept	-1.06	0.184	1.000	-5.776	< 0.001	
$\log_{10}$ Area (km²)			1.933	13.852	< 0.001	
MLSR			1.000	11.596	0.001	
-1 x $\log_{10}$ SLMP			1.000	13.384	0.001	
Variation in precipitation			1.656	7.603	0.001	
Temperature range (°C)			1.951	7.447	0.001	
**Ferns (genus)**						0.18
Intercept	0.271	0.067	1.000	4.025	< 0.001	
Elevation range (m)			1.859	5.521	0.005	
$\log_{10}$ Area (km²)			1.000	4.783	0.030	
MLSR			1.894	8.016	< 0.001	
Precipitation (mm)			1.000	9.083	0.003	
Temperature (°C)			1.902	12.679	< 0.001	
Variation in precipitation			1.000	5.376	0.021	

Supplementary Table S4. Variable importance estimated from all possible multi-predictor Generalized Additive Models for the standardized effect size of mean pairwise phylogenetic distance (MPD_{es}) of angiosperms, palms and ferns on islands in dependence on environmental predictors. Importance was assessed as the sum of Akaike's Information Criterion corrected for small sampling sizes (AIC_c) weights of all models in which a variable was included. Apart from island predictor variables, all candidate models also included spatial eigenvectors to account for spatial autocorrelation. Angiosperm MPD_{es} was calculated based on a dated family-level phylogeny, and palm MPD_{es} based on a dated genus-level phylogeny. Fern MPD_{es} was based on dated phylogenies at both family and genus levels (n = 363 islands for all angiosperms, n = 71 islands for palms and n = 234 islands for ferns). Columns Disp (dispersal filtering), Spec (*in-situ* speciation) and Env (environmental filtering) indicate which hypothesized process the variables relate to. Values larger than 0.8 are printed in bold.

	**Process (Fig. 1)**			**Family-level phylogeny**		**Genus-level phylogeny**	
**Variable**	**Disp**	**Spec**	**Env**	**Angiosperms**	**Ferns**	**Palms**	**Ferns**
Mainland species richness (MLSR)	X			**0.93**	**0.99**	0.3	**1**
Geologic history (fragment, shelf, oceanic)	X	X		0.7	0.59	0.67	0.46
-1 x $\log_{10}$ Surrounding landmass proportion (SLMP)	X	X		**0.93**	0.78	**0.84**	**1**
$\log_{10}$ Island area (km²)		X		**1**	**0.97**	**1**	**1**
Elevation range (m)		X	X	**0.97**	**1**	**0.85**	**1**
Annual mean temperature (°C)			X	0.4	**0.95**	**0.94**	0.5
Temperature seasonality (annual range; °C)			X	**0.99**	0.36	0.35	**0.85**
Annual precipitation (mm)			X	**1**	**0.81**	0.2	0.31
Precipitation seasonality (coefficient of variation)			X	0.31	0.44	**0.88**	0.26
$\log_{10}$ Late Quaternary climate change velocity in temperature (m y^{-1}; CCVT)			X	0.67	**0.94**	0.3	**1**

Supplementary Table S5. Best Generalized Additive Models based on Akaike's Information Criterion corrected for small sampling sizes of the relationships of the standardized effect size of mean pairwise phylogenetic distance (MPD_{es}) of angiosperms, palms and ferns with environmental factors on islands. Models included spatial eigenvectors to account for spatial autocorrelation. For angiosperms, MPD_{es} was calculated based on a dated family-level phylogeny, and for palms based on a dated genus-level phylogeny. For comparison, MPD_{es} of ferns was calculated using phylogenies at both family and genus levels. (n = 363 islands for all angiosperms, n = 71 islands for palms and n = 234 islands for ferns). Estimates for factor levels of geologic history are given relative to continental fragments. R^2 is a partial R^2 for the predictor variables, removing the effect of the spatial eigenvectors; edf = effective degrees of freedom; column t/F-value contains t-values in case of intercepts and linear effects (edf =1) and F-values in case of smooth terms (edf > 1); MLSR = Mainland species richness, SLMP = surrounding landmass proportion, CCVT = Late Quaternary climate change velocity.

Term	Estimate	Std. error	edf	t/F-value	p-value	R^2
**Angiosperms**						0.37
Intercept	-1.559	0.248	1.000	-6.290	< 0.001	
Geologic history (oceanic)	0.278	0.288	1.000	0.966	0.335	
Geologic history (shelf)	0.819	0.351	1.000	2.330	0.020	
Elevation range (m)			1.000	7.955	0.005	
log_{10} Area (km^2)			1.000	18.066	< 0.001	
log_{10} CCVT (m/y)			1.000	3.869	0.050	
MLSR			1.882	3.893	0.022	
Precipitation (mm)			1.935	18.350	< 0.001	
-1 x log_{10} SLMP			1.660	5.064	0.008	
Temperature range (°C)			1.940	7.226	0.001	
**Ferns (family)**						0.25
Intercept	1.030	0.234	1.000	4.406	< 0.001	
Geologic history (oceanic)	-0.617	0.279	1.000	-2.212	0.028	
Geologic history (shelf)	-0.525	0.321	1.000	-1.634	0.104	
Elevation range (m)			1.862	8.794	< 0.001	
log_{10} Area (km^2)			1.384	6.389	0.005	
log_{10} CCVT (m/y)			1.937	6.154	0.003	
MLSR			1.550	7.668	0.001	
Precipitation (mm)			1.000	5.789	0.017	
-1 x log_{10} SLMP			1.615	2.838	0.065	
Temperature (°C)			1.000	13.984	< 0.001	
Variation in precipitation			1.000	2.138	0.145	
**Palms**						0.59
Intercept	-3.027	0.791	1.000	-3.828	< 0.001	
Geologic history (oceanic)	0.269	1.152	1.000	0.233	0.816	
Geologic history (shelf)	4.918	1.913	1.000	2.571	0.013	
Elevation range (m)			1.705	4.959	0.011	
log_{10} Area (km^2)			2.000	21.465	< 0.001	
-1 x log_{10} SLMP			1.000	5.648	0.021	
Temperature (°C)			1.931	5.746	0.005	
Variation in precipitation			1.000	7.352	0.009	
**Ferns (genus)**						0.24
Intercept	0.585	0.078	1.000	7.545	< 0.001	
Elevation range (m)			1.867	8.278	< 0.001	
log_{10} Area (km^2)			1.000	35.587	< 0.001	
log_{10} CCVT (m/y)			1.948	16.175	< 0.001	
MLSR			1.707	14.465	< 0.001	
-1 x log_{10} SLMP			1.935	9.101	< 0.001	
Temperature (°C)			1.000	2.720	0.101	
Temperature range (°C)			1.288	4.856	0.017	

Supplementary Table S6. Pearson correlations among predictor variables used to explain phylogenetic structure of island floras (n = 393). Correlations with island age were calculated for a subset of n = 202 oceanic islands with information on the age of emergence. Coefficients and p-values were corrected for spatial autocorrelation following ref. 47. MLSR = mainland species richness, -SLMP = -1 x log_{10} surrounding landmass proportion, Area = log_{10} island area (km^2), Elev = elevation range (m), Temp = annual mean temperature (°C), varT = annual temperature range (°C), Prec = annual precipitation (mm), varP = variation in monthly precipitation, CCVT = log_{10} Late Quaternary climate change velocity (m/y), Age = island age (my). Coefficients larger than 0.7 are printed in bold. Significance: * p < 0.05, ** p < 0.01, *** p < 0.001.

	MLSR	- SLMP	Area	Elev	Temp	varT	Prec	varP	CCVT
**- SLMP**	0.09								
**Area**	0.04	-0.08							
**Elev**	0.01	-0.01	**0.78*** **						
**Temp**	0.55***	0.23	-0.10	-0.07					
**varT**	-0.30*	**-0.70*** **	-0.02	-0.04	-0.55**				
**Prec**	0.22	0.43***	0.28***	0.43***	0.19	-0.29**			
**varP**	-0.07	-0.32*	-0.17*	-0.18*	0.31*	0.05	-0.48***		
**CCVT**	-0.22*	-0.34**	-0.48***	-0.48***	-0.43***	0.47***	-0.26**	-0.07	
**Age**	0.12	-0.12	0.14	-0.14	0.20*	-0.26***	-0.14	0.16*	-0.08

Supplementary Table S7. Pearson correlations among phylogenetic community metrics calculated with a global island species pool and three different regional species pool delineations (standardized effect size of phylogenetic diversity (PD_{es}) and mean pairwise phylogenetic distance (MPD_{es})). Regional species pools include all species of all islands that 1) belong to a particular floristic realm after Takhtajan[48] (Regions; Australis included in Holantarctis), 2) belong to a major ocean basin (Oceans; i.e. Pacific, Atlantic, Indian Ocean), or 3) are located within 10,000 km around each target island[49] (Circular; see Supplementary Fig. S9). Coefficients and p-values were corrected for spatial autocorrelation[47]. Significance: * p < 0.05, ** p < 0.01, *** p < 0.001.

Metric	Taxon	Regions	Oceans	Circular
**PD_{es}**	**Angiosperms**	0.87 ***	0.96 ***	0.96 ***
	**Ferns (family)**	0.93 ***	0.98 ***	0.99 ***
	**Ferns (genus)**	0.95 ***	0.97 ***	0.99 ***
	**Palms**	0.96 ***	0.94 ***	0.97 ***
**MPD_{es}**	**Angiosperms**	0.94 ***	0.90 ***	0.91 ***
	**Ferns (family)**	0.86 ***	0.99 ***	0.99 ***
	**Ferns (genus)**	0.87 ***	0.99 ***	0.99 ***
	**Palms**	0.97 ***	0.97 ***	0.97 ***

Supplementary Table S8. Variation explained by Generalized Additive Models (GAM) of the standardized effect size of phylogenetic diversity (PD_{es}) and mean pairwise phylogenetic distance (MPD_{es}) of angiosperms overall, palms and ferns on islands based on a global island species pool (Global) and three different regional species pool delineations (Regions, Oceans, Circular) as response variables and environmental predictor variables. Results are shown for the best models retained after a model selection procedure based on Akaike's Information Criterion corrected for small sampling sizes (see methods for the set of environmental predictors included). In addition to the environmental predictors, the models included spatial eigenvectors to account for spatial autocorrelation. Here, we report pseudo R^2-values derived from linear models of observed vs. predicted values from the GAMs, disregarding the spatial filters in the predictions, to estimate variation explained by environmental predictors alone. Regional species pools included all species of all islands that 1) belong to a particular floristic realm after Takhtajan[48] (Regions; Australis included in Holantarctis), 2) belong to a major ocean basin (Oceans; i.e. Pacific, Atlantic, Indian Ocean), or 3) are located within 10,000 km around each target island[49] (Circular; see Supplementary Fig. S9).

Metric	Taxon	Global	Regions	Oceans	Circular
**PD_{es}**	**Angiosperms**	48.6	34.3	45.7	48.3
	**Ferns (family)**	15.1	16.7	13.5	11.9
	**Ferns (genus)**	18.1	18.8	18.8	19.7
	**Palms**	51.6	54.6	53.4	52.0
**MPD_{es}**	**Angiosperms**	36.8	33.2	34.3	39.7
	**Ferns (family)**	25.3	15.6	24.0	17.0
	**Ferns (genus)**	24.1	13.0	26.8	17.2
	**Palms**	58.8	52.4	48.7	47.5

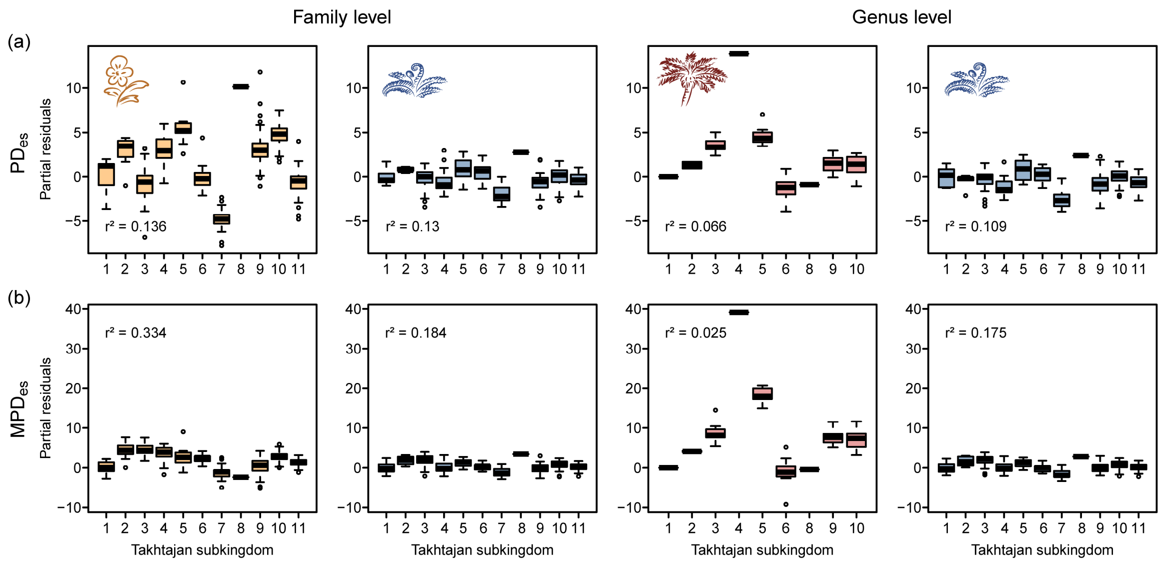

Supplementary Figure S1. Partial residual plots for Takhtajan's floristic subkingdoms derived from averaged Generalized Additive Models of the relationships of the standardized effect sizes of (a) phylogenetic diversity (PD_{es}) and (b) mean pairwise phylogenetic distance (MPD_{es}) of angiosperms, palms and ferns with environmental factors on islands. In addition to Takhtajan's floristic subkingdoms[48], models included ten environmental predictors as well as spatial eigenvectors to account for spatial autocorrelation. PD_{es} and MPD_{es} were calculated based on a dated family-level phylogeny for angiosperms (orange) and based on a dated genus-level phylogeny for palms (red). Metrics for ferns (blue) were calculated based on a dated family-level phylogeny for comparison with angiosperms (column 2), and based on a dated genus-level phylogeny for comparison with palms (column 4). Only islands with at least two species of the focal group were included in models (n = 363 for all angiosperms, n = 71 for palms only and n = 234 for ferns). Takhtajan's floristic subkingdoms: 1 = African, 2 = Australian, 3 = Boreal, 4 = Holantarctic, 5 = Indomalesian, 6 = Madagascan, 7 = Madrean, 8 = Neocaledonian, 9 = Neotropical, 10 = Polynesian, 11 = Tethyan.

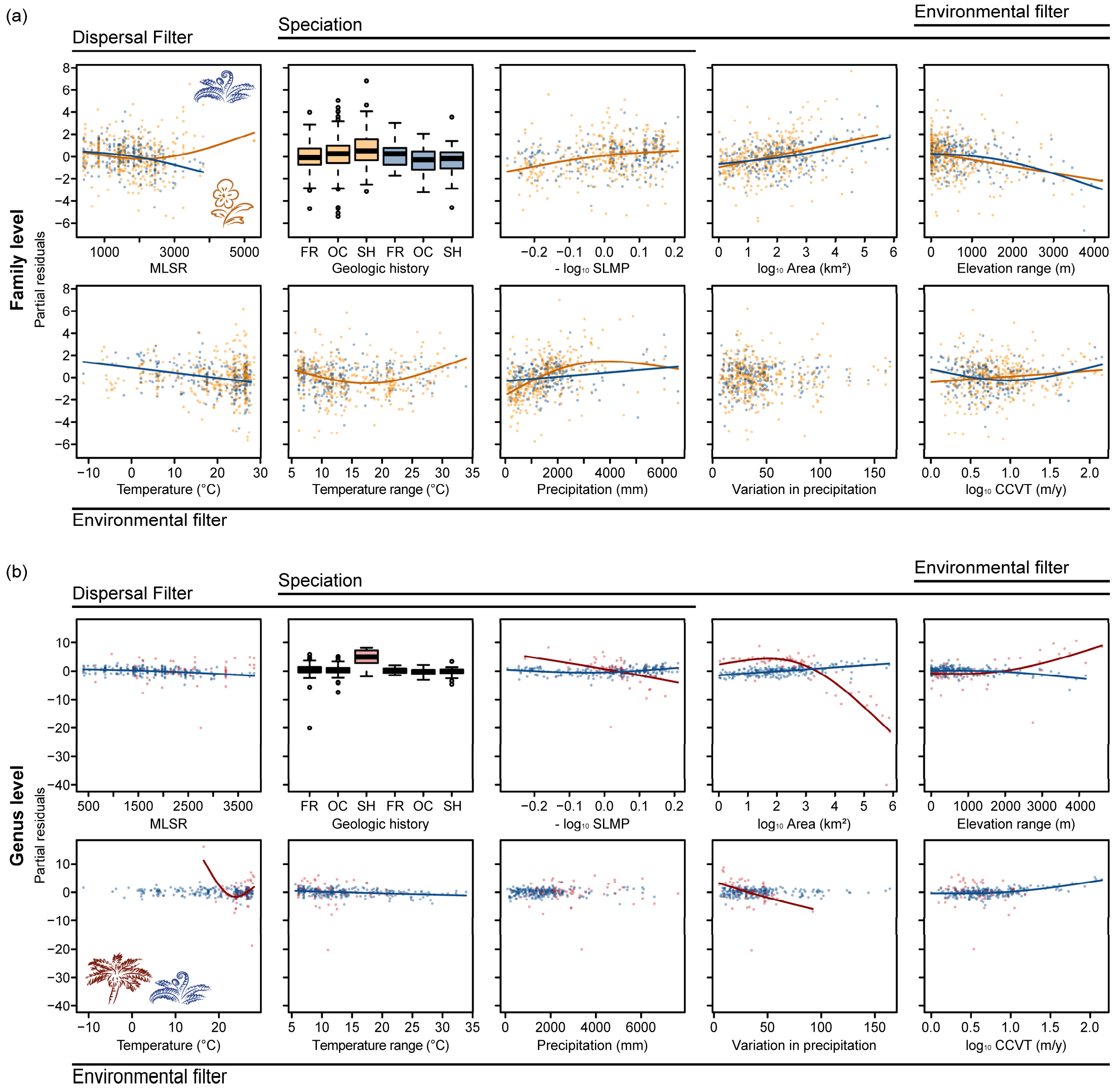

Supplementary Figure S2. Partial residual plots from averaged Generalized Additive Models of the relationships of the standardized effect size of mean pairwise phylogenetic distance (MPD_{es}) of angiosperms, palms and ferns with environmental factors on islands. Models included spatial eigenvectors to account for spatial autocorrelation. Regression lines are shown if the variable was significant in the averaged model. In (a), MPD_{es} was based on dated family-level phylogenies of angiosperms (orange) and ferns (blue). In (b), MPD_{es} was based on dated genus-level phylogenies of palms (red) and ferns (blue). Only islands with at least two species of the focal group are shown (n = 363 for all angiosperms, n = 71 for palms and n = 234 for ferns). MLSR = Mainland species richness, SLMP = surrounding landmass proportion, CCVT = Late Quaternary climate change velocity; Geologic island types: FR = continental fragment, OC = oceanic island (volcanic islands, atolls, uplifted sea floor), SH = continental shelf islands.

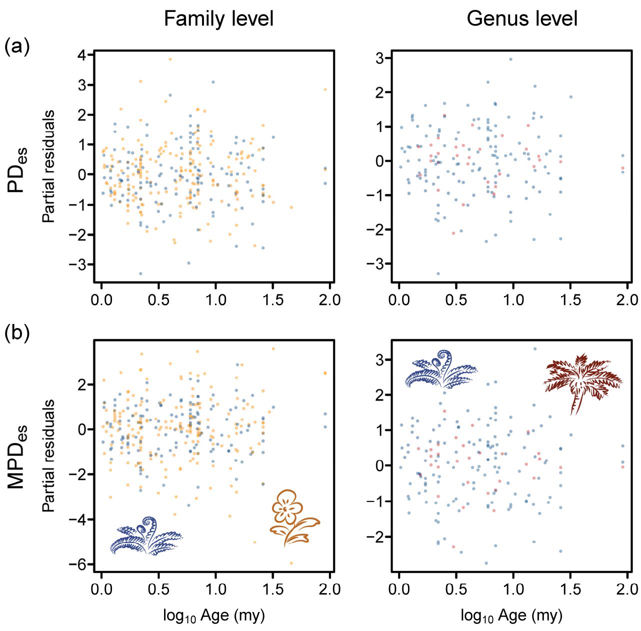

Supplementary Figure S3. Partial residual plots for island geologic age derived from averaged Generalized Additive Models of the relationships of the standardized effect sizes of (a) phylogenetic diversity (PD_{es}) and (b) mean pairwise phylogenetic distance (MPD_{es}) of angiosperms, palms and ferns with environmental factors on islands. In addition to island age, the full models included ten environmental predictors and spatial eigenvectors to account for spatial autocorrelation. The effect of island age was not significant in any averaged model ($p > 0.05$). Regression lines are therefore not plotted. PD_{es} and MPD_{es} were calculated based on a dated family-level phylogeny for angiosperms (orange) and based on a dated genus-level phylogeny for palms (red). Metrics for ferns (blue) were calculated based on a dated family-level phylogeny for comparison with angiosperms (column 1), and based on a dated genus-level phylogeny for comparison with palms (column 2). Only islands with at least two species of the focal group and with information on island age were included in models (n = 187 islands for all angiosperms, n = 31 islands for palms only and n = 138 islands for ferns).

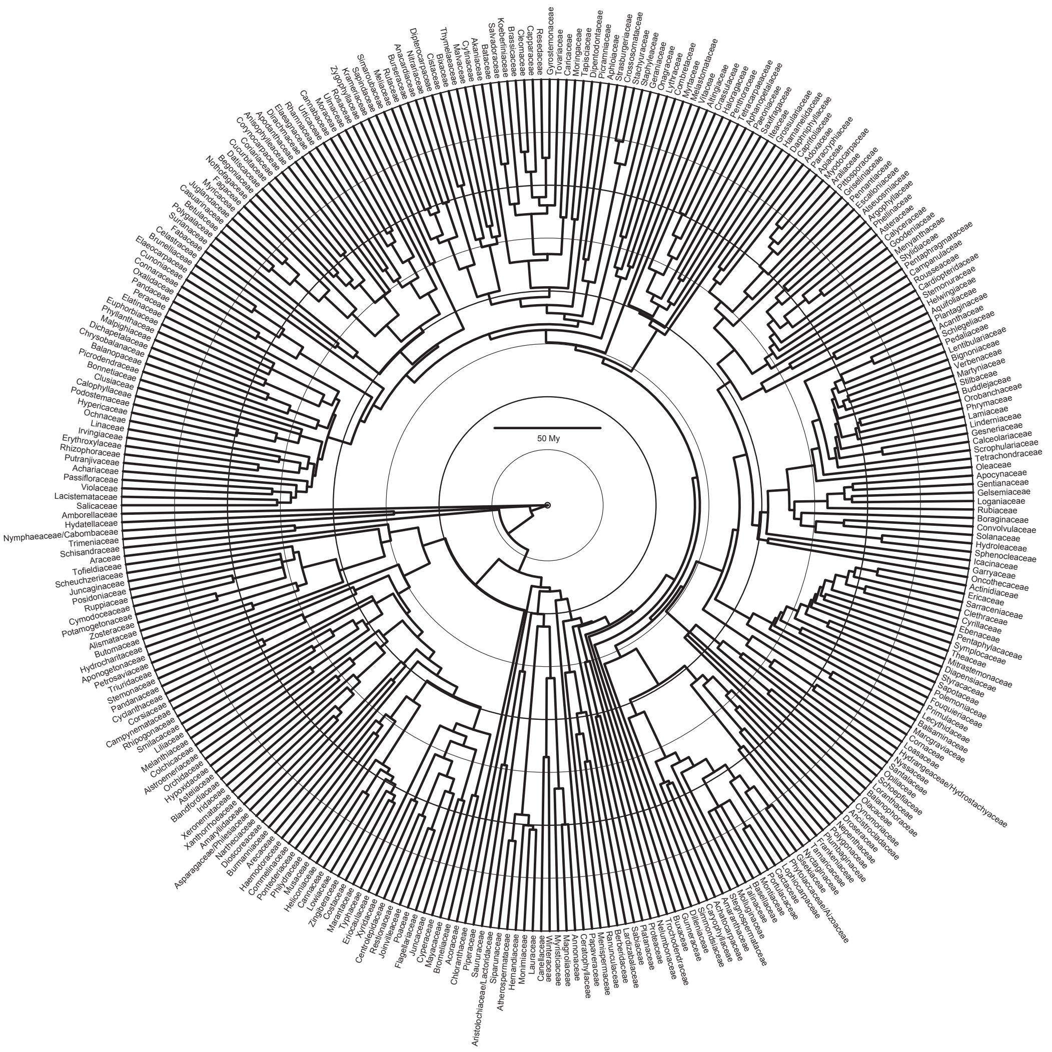

Supplementary Figure S4. Dated angiosperm phylogeny used to analyse the phylogenetic structure of island floras. The original phylogeny[25], including 560 angiosperm species from 335 families, was pruned to family level and to only include families present in the considered island floras (315 families and merged groups). Five pairs of families that would otherwise not be monophyletic were merged (names at tree tips separated by slash). 60 families missing from the phylogeny were manually added according to the phylogeny from ref. 26, the angiosperm phylogeny group[24] classification III and information on the angiosperm phylogeny website[27] (see Supplementary Methods S1 for details).

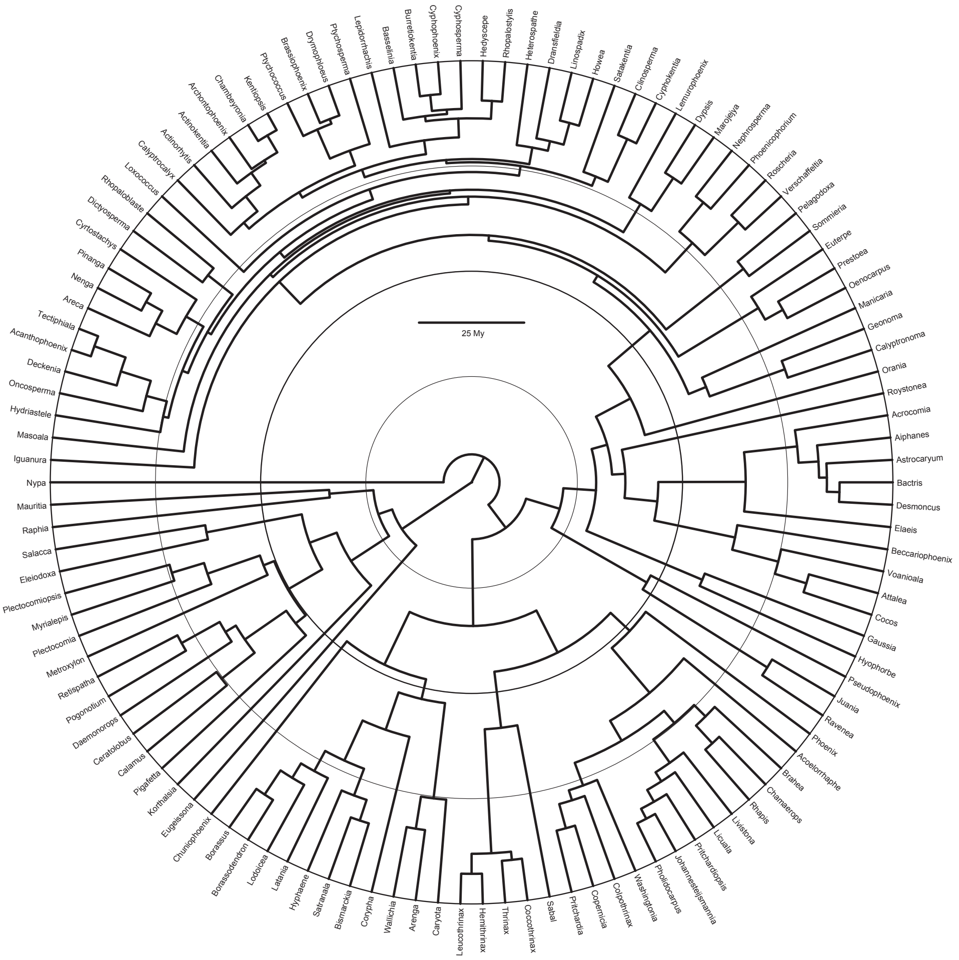

Supplementary Figure S5. Dated palm phylogeny used to analyse the phylogenetic structure of island floras. The original phylogeny[28] was pruned to genus level and only includes genera present in the considered island floras (118 genera; see Supplementary Methods S1 for details).

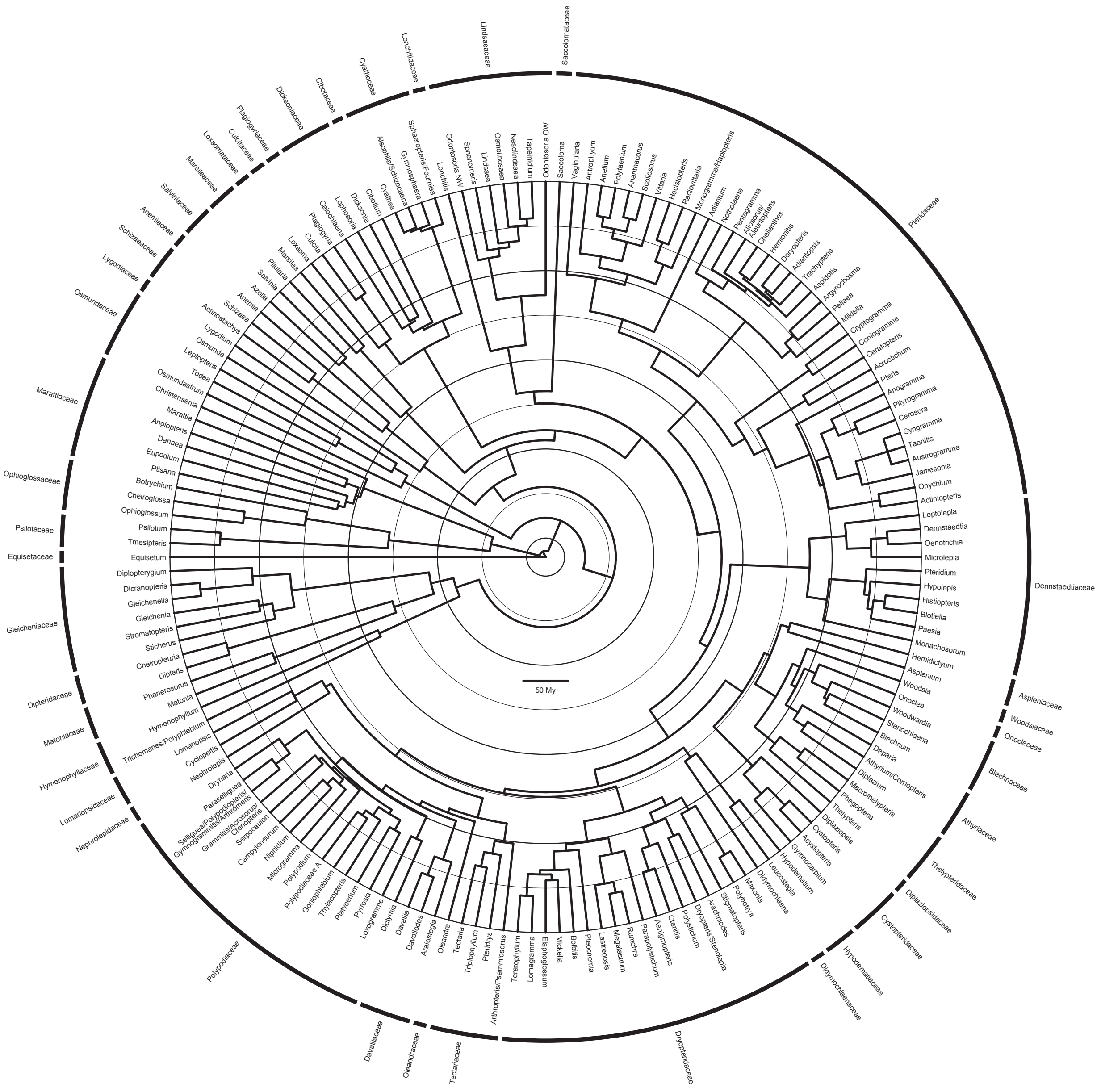

Supplementary Figure S6. Dated fern phylogeny used to analyse the phylogenetic structure of island floras. The original phylogeny including 1,118 fern species was pruned to genus level and to only include genera present on the considered islands (168 genera, 42 families). 25 genera were placed manually to the tree according to literature information (see Supplementary Methods S1 for details). The genus *Odontosoria* was split into an old world clade (*Odontosoria* OW) and a new world clade (*Odontosoria* NW). Genus names separated by slashes and the label "Polypodiaceae A" indicate groups of merged genera. The outside ring of lines and names indicates how fern families were delimited in the family-level phylogeny.

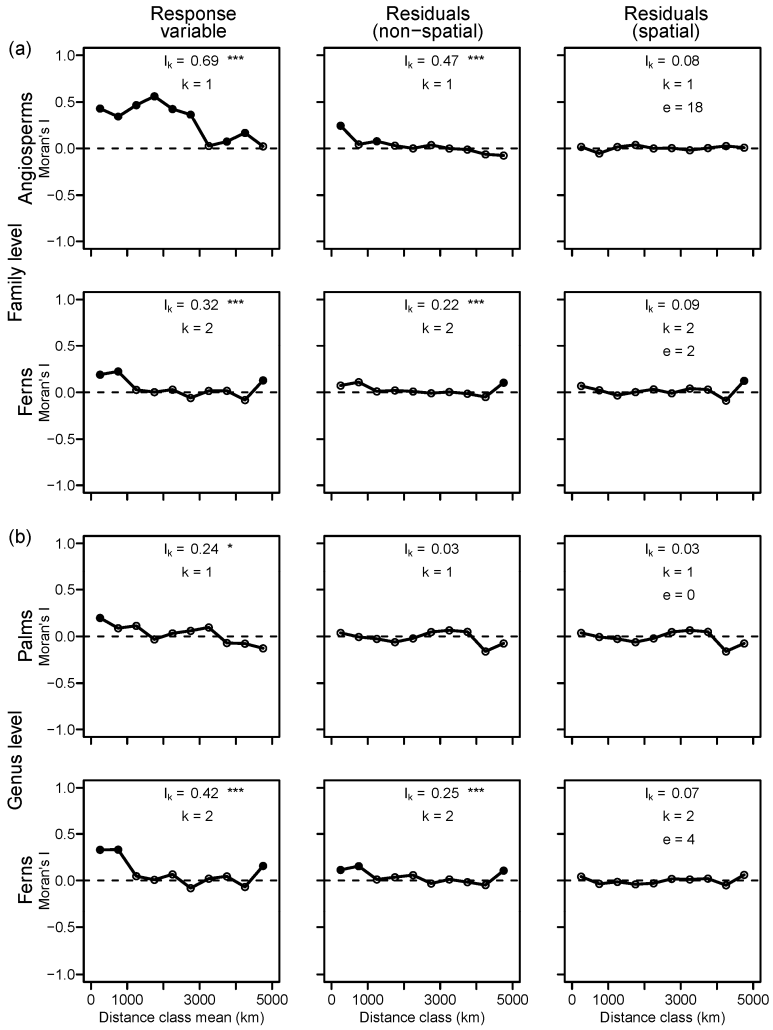

Supplementary Figure S7. Moran's *I* correlograms of spatial autocorrelation for the standardized effect size of phylogenetic diversity (PD_{es}; Response variable) of angiosperms, palms and ferns on islands; residuals from the best Generalized Additive Models of PD_{es} in dependence on environmental predictors (Residuals (non-spatial)); and residuals from best spatial models (Residuals (spatial)) including a set of *e* spatial eigenvectors to reduce spatial autocorrelation (see Supplementary Methods S2 for details). In (a), PD_{es} was calculated based on dated family-level phylogenies of angiosperms and ferns. In (b), PD_{es} was calculated based on dated genus-level phylogenies of palms and ferns. Filled circles indicate spatial autocorrelation significant at $p < 0.05$; open circles indicate non-significant autocorrelation. I_k values are Moran's *I* values based on row standardized neighbourhood lists of k neighbours per island. We show the highest Moran's *I* value out of values for k = 1 to k = 25. Significance of I_k: * $p < 0.05$, *** $p < 0.001$.

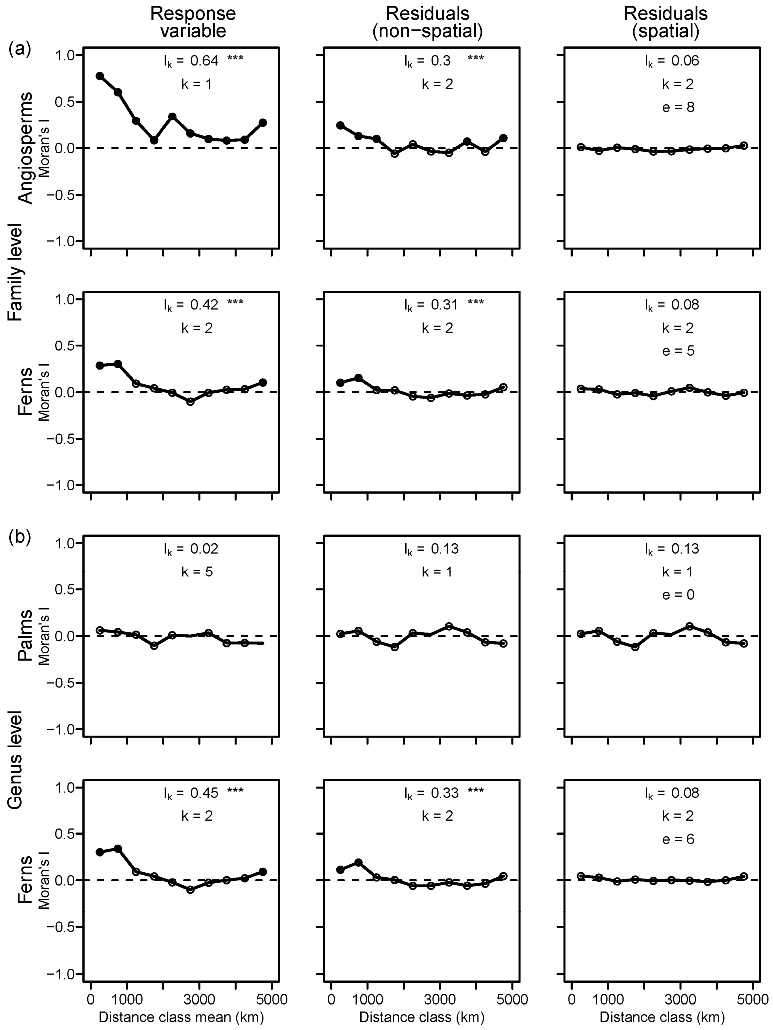

Supplementary Figure S8. Moran's *I* correlograms of spatial autocorrelation for the standardized effect size of mean pairwise phylogenetic distance (MPD_{es}; Response variable) of angiosperms, palms and ferns on islands; residuals from the best Generalized Additive Models of MPD_{es} in dependence on environmental predictors (Residuals (non-spatial)); and residuals from bes spatial models (Residuals (spatial)) including a set of e spatial eigenvectors to reduce spatial autocorrelation (see Supplementary Methods S2 for details). In (a), MPD_{es} was calculated based on dated family-level phylogenies of angiosperms and ferns. In (b), MPD_{es} was calculated based on dated genus-level phylogenies of palms and ferns. Filled circles indicate spatial autocorrelation significant at $p < 0.05$ in contrast to non-significant autocorrelation (open circles). I_k values are Moran's *I* values based on row standardized neighbourhood lists of k neighbours per island (I_k). We show the highest Moran's *I* value out of values for k = 1 to k = 25. Significance of I_k: * $p < 0.05$, *** $p < 0.001$.

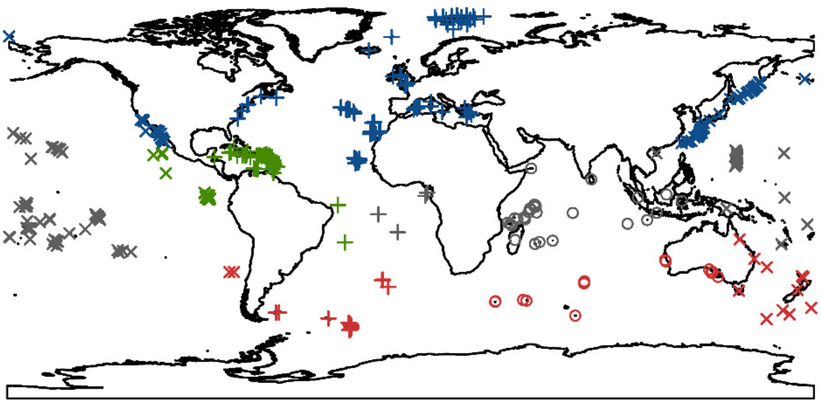

Supplementary Figure S9. Three different delineations of regional species pools used in sensitivity analyses. Colours indicate floristic realms after Takhtajan[48] (Australis included in Holantarctis): blue = Holarctis, green = Neotropis, grey = Palaeotropis, red = Holantarctis (including Australis); Symbols indicate regions delimited after the major ocean basins: x = Pacific, + = Atlantic, ○ = Indian. In addition, we used all plants on all islands within 10,000 km around each target island as circular regional species pools[49]. The map was created in R[50].

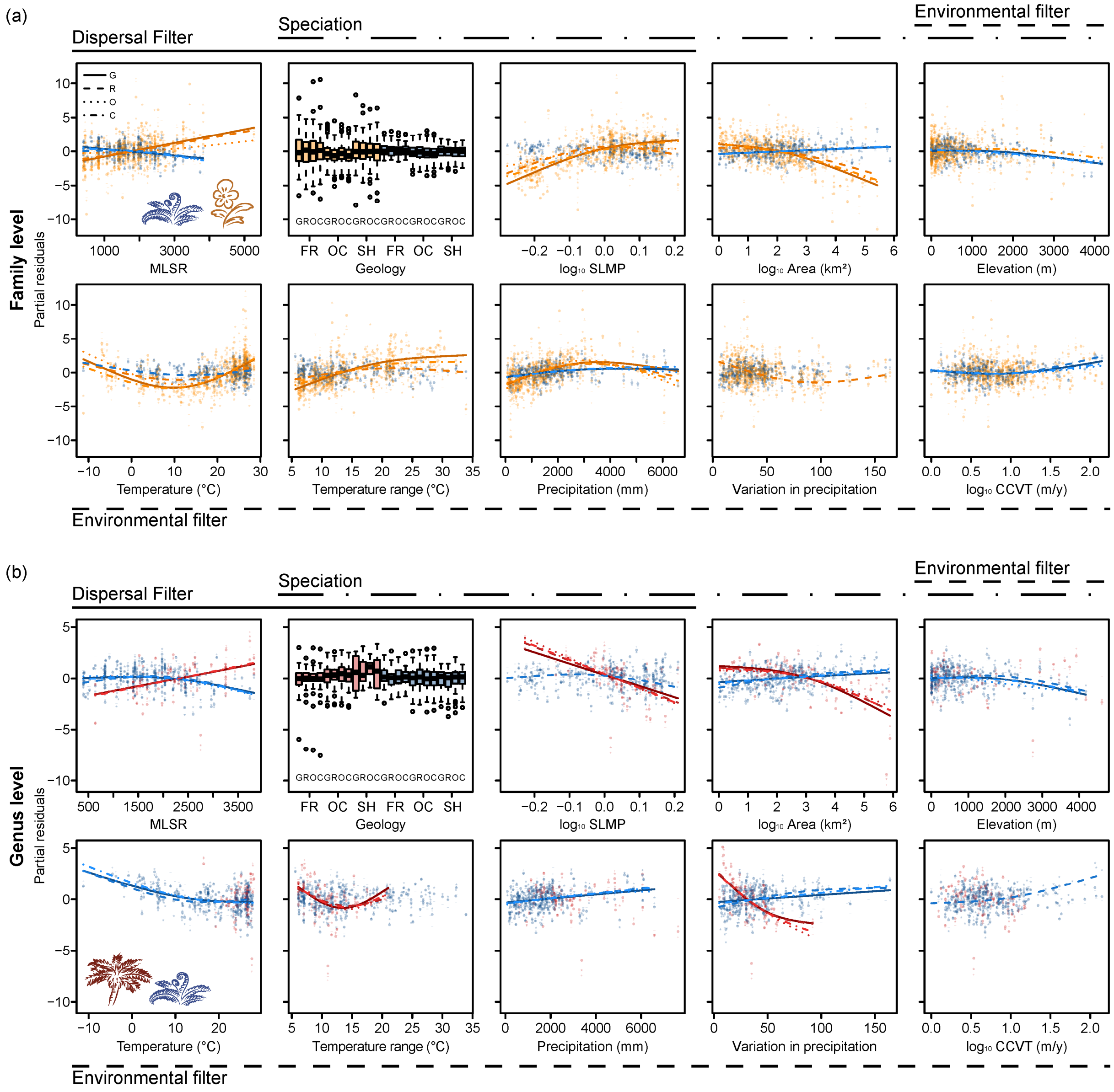

Supplementary Figure S10. Species pool sensitivity analyses for environmental predictors of phylogenetic structure in island floras. Equivalent to Fig. 4, partial residual plots from averaged Generalized Additive Models show the standardized effect size of phylogenetic diversity (PD_{es}) of angiosperms overall, palms and ferns as a function of environmental predictors. Here, however, results are shown based on different regional species pool delineations in addition to the global island species pool (denoted G; solid lines, symbol ●). Regional species pools include all species of all islands that 1) belong to a particular floristic realm after Takhtajan[48] (denoted R; dashed lines; symbol *; Australis included in Holantarctis), 2) belong to a major ocean basin (denoted O; dashed lines; symbol -; i.e. Pacific, Atlantic, Indian Ocean), or 3) are located within 10,000 km around each target island[49] (denoted C; dashed lines; symbol +; see Supplementary Fig. S9). Models included spatial eigenvectors to account for spatial autocorrelation. Regression lines are shown if the variable was significant in the

averaged model for the given species pool delineation. In (a), PD_{es} was based on dated family-level phylogenies of angiosperms (orange) and ferns (blue). In (b), PD_{es} was based on dated genus-level phylogenies of palms (red) and ferns (blue). Only islands with at least two species of the focal group are shown (363 islands for all angiosperms, 71 for palms and 234 for ferns). MLSR = Mainland species richness, SLMP = surrounding landmass proportion, CCVT = Late Quaternary climate change velocity; Geologic island types: FR = continental fragment, OC = oceanic island, SH = continental shelf islands.

Supplementary References S1. Literature used to compile the global dataset of angiosperm, palm and fern species composition on 393 islands worldwide.

Acevedo-Rodríguez, P. & Strong, M. T. *Catalogue of the seed plants of the West Indies Website.* (2007) Available at: http://botany.si.edu/antilles/WestIndies/index.htm. (Date of access: 1 March 2011).

Alves, R. J. V. *Ilha da Trindade e Arquipélago Martin Vaz: um ensaio geobotânico*. (Serviço de Documentação da Marinha, Rio de Janeiro, Brasil, 1998).

Arechavaleta, M., Zurita, N., Marrero, M. C. & Martín, J. L. *Lista preliminar de especies silvestres de Cabo Verde (hongos, plantas y animales terrestres)*. (Consejería de Medio Ambiente y Ordenación Territorial, Gobierno de Canarias, 2005).

Arechavaleta, M., Rodríguez, S., Zurita, N. & García, A. *Lista de especies silvestres de Canarias. Hongos, plantas y animales terrestres*. (Consejería de Medio Ambiente y Ordenación Territorial, Gobierno de Canarias, 2009).

Ashmole, P. & Ashmole, M. *St Helena and Ascension Island: a natural history*. (Anthony Nelson Ltd, 2000).

Athens, J. S., Blinn, D. W. & Ward, J. V. Vegetation history of Laysan Island, Northwestern Hawaiian Islands. *Pac. Sci.* **61**, 17-37 (2007).

Baker, M. L. & Duretto, M. F. *A census of the vascular plants of Tasmania*. (Tasmanian Herbarium, Tasmanian Museum and Art Gallery, 2011).

Barker, W. R., Barker, R. M., Jessop, J. P. & Vonow, H. P. Census of South Australian vascular plants. *Journal of the Adelaide Botanic Gardens Supplement* **1**, 1–396 (2005).

Borges, P. A. V. *et al. A list of the terrestrial fauna (Mollusca and Arthropoda) and flora (Bryophyta, Pteridophyta and Spermatophyta) from the Azores*. (Direcção Regional do Ambiente and Universidade dos Açores, 2005).

Borges, P. A. V. *et al. Listagem dos fungos, flora e fauna terrestres dos arquipélagos da Madeira e Selvagens*. (Direcção Regional do Ambiente da Madeira and Universidade dos Açores, 2008).

Bowdoin Scientific Station. *Vascular plants of Kent Island.* (2011) Available at: http://www.bowdoin.edu/kent-island/species/plants.shtml. (Date of access: 14 September 2011).

Brofas, G., Karetsos, G., Panitsa, M. & Theocharopoulos, M. The flora and vegetation of Gyali Island, SE Aegean, Greece. *Willdenowia* **31**, 51-70 (2001).

Broughton, D. A. & McAdam, J. H. A checklist of the native vascular flora of the Falkland Islands (Islas Malvinas): new information on the species present, their ecology, status and distribution. *J. Torrey Bot. Soc.* **132**, 115-148 (2005).

Burton, R. M. A check-list and evaluation of the flora of Nisyros (Dodecanese, Greece). *Willdenowia* **20**, 15-38 (1991).

Butler, B. J., Barclay, J. S. & Fisher, J. P. Plant communities and flora of Robins Island (Long Island), New York. *J. Torrey Bot. Soc.* **126**, 63-76 (1999).

Byrd, G. V. Vascular vegetation of Buldir Island, Aleutian Islands, Alaska, compared to another Aleutian Island. *Arctic* **37**, 37-48 (1984).

CARMABI. *Dutch Caribbean Biodiversity Explorer.* (2009) Available at: http://www.dcbiodata.net/explorer/home. (Date of access: 24 June 2011).

Case, T. J., Cody, M. L. & Ezcurra, E. *A new island biogeography of the Sea of Cortés*. (Oxford University Press, 2002).

Chernyaeva, A. M. Flora of Onekotan Island. *Bulletin of Main Botanical Garden* **87**, 21-29 (1973).

Christmas Island National Park. *Third Christmas Island national park management plan*. (Parks Australia North, Christmas Island, Australia, 2002).

Christodoulakis, D. The flora of Ikaria (Greece, E. Aegean Islands). *Phyton* **36**, 63-91 (1996).

Christophersen, E. Vascular plants of Johnston and Wake Islands. *Occasional Papers of the Bernice Pauahi Bishop Museum of Polynesian* **9**, 1-20 (1931).

Conti, F., Abbate, G., Alessandrini, A. & Blasi, C. *Annotated Checklist of the Italian Vascular Flora*. (Palombi Editori, Roma, 2005).

Convey, P., Lewis Smith, R. I., Hodgson, D. A. & Peat, H. J. The flora of the South Sandwich Islands, with particular reference to the influence of geothermal heating. *J. Biogeogr.* **27**, 1279-1295 (2000).

Cronk, Q. C. B. The past and present vegetation of St Helena. *J. Biogeogr.* **16**, 47-64 (1989).

D'Arcy, W. G. The island of Anegada and its flora. *Atoll Res. Bull.* **139**, 1-21 (1971).

de Lange, P. J. & Cameron, E. K. The vascular flora of Aorangi Island, Poor Knights Islands, northern New Zealand. *N. Z. J. Bot.* **37**, 433-468 (1999).

de Miranda Freitas, A. M. *A Flora Fanerogâmica Atual do Arquipélago de Fernando de Noronha - Brasil*. (Universidade Estadual, 2007).

Directorate of Wrangel Island Reserve. *Natural System of Wrangel Island Reserve*. (The World Heritage Committee, 2003).

Dowhan, J. J. & Rozsa, R. Flora of Fire Island, Suffolk County, New York. *B. Torrey Bot. Club* **116**, 265-282 (1989).

Du Puy, D. J. *Christmas Island: species lists.* (1993) Available at: http://www.anbg.gov.au/abrs/online-resources/flora/stddisplay.xsql?pnid=54674. (Date of access: 6 April 2011).

Egorova, E. M. Flora of Shiashkotan Island. *Bulletin of the Main Botanical Garden* **54**, 114-120 (1964).

Esler, A. E. Botanical features of the Mokohinau Islands. *TANE* **24**, 187-197 (1978).

Exell, A. W. *Catalogue of the vascular plants of S. Tome (with Principe and Annobon)*. (Trustees of the British Museum, 1944).

Florence, J., Chevillotte, H., Ollier, C. & Meyer, J.-Y. *Base de données botaniques Nadeaud de l'Herbier de la Polynésie française (PAP).* (2007) Available at: http://www.herbier-tahiti.pf. (Date of access: 1 July 2011).

Fosberg, F. R. Flora of Johnston Island, Central Pacific. *Pac. Sci.* **3**, 338-339 (1949).

Fosberg, F. R., Renvoize, S. A. & Townsend, C. C. *The flora of Aldabra and neighbouring islands*. (HMSO, 1980).

Fosberg, F. R., Stoddart, D. R., Sachet, M.-H. & Spellman, D. L. Plants of the Belize cays. *Atoll Res. Bull.* **258**, 1-77 (1982).

Fosberg, F. R. & Sachet, M. H. Flora of Maupiti, Society Islands. *Atoll Res. Bull.* **294**, 1-70 (1987).

Franklin, J., Keppel, G. & Whistler, W. A. The vegetation and flora of Lakeba, Nayau and Aiwa islands, central Lau Group, Fiji. *Micronesica* **40**, 169-225 (2008).

Gabrielsen, G. W., Brekke, B., Alsos, I. G. & Hansen, J. R. *Natur-og kulturmiljøet på Jan Mayen*. Vol. 144 1-127 (Norsk Polarinstitutt, 1997).

Gage, S., Joneson, S. L., Barkalov, V. Y., Eremenko, N. A. & Takahashi, H. A newly compiled checklist of the vascular plants of the Habomais, the Little Kurils. *Bulletin of the Hokkaido University Museum* **3**, 67-91 (2006).

Gerlach, J. The biodiversity of the granitic islands of Seychelles. *Phelsuma* **11 (Supplement A)**, 1-47 (2003).

Green, P. S. *Lord Howe Island: species lists.* (1994) Available at: http://www.environment.gov.au/biodiversity/abrs/online-resources/flora/49/index.html. (Date of access: 6 April 2011).

Greene, S. & Walton, D. An annotated check list of the sub-Antarctic and Antarctic vascular flora. *Polar Rec.* **17**, 473-484 (1975).

Hill, S. R. An annotated checklist of the vascular flora of Assateague Island (Maryland and Virginia). *Castanea* **51**, 265-305 (1986).

Hill, M. J. *Biodiversity surveys and conservation potential of inner Seychelles islands*. (Smithsonian Institution, 2002).

Hnatiuk, R. J. *Subantarctic Islands: species lists.* (1993) Available at: http://www.environment.gov.au/biodiversity/abrs/online-resources/flora/50/index.html. (Date of access: 7 April 2011).

Iwatsuki, K., Yamazaki, T., Boufford, D. & Ohba, H. *Flora of Japan, Vol. 1 - Pteridophyta and Gymnospermae*. (Kodansha, 1995).

Jackes, B. R. *Plants of Magnetic Island*. 3 edn (James Cook University, 2010).

Jaramillo Díaz, P. & Guézou, A. *CDF checklist of Galapagos vascular plants.* (2011) Available at: http://www.darwinfoundation.org/datazone/checklists/vascular-plants/. (Date of access: 25 February 2011).

Johnson, P. N. & Campbell, D. J. Vascular plants of the Auckland Islands. *N. Z. J. Bot.* **13**, 665-720 (1975).

Johnston, I. M. The flora of the Revillagigedo Islands. *Proc. Calif. Acad. Sci.* **20**, 9-104 (1931).

Junak, S., Philbrick, R., Chaney, S. & Clark, R. *A checklist of vascular plants of Channel Islands National Park*. 2 edn (Southwest Parks and Monuments Association, 1997).

Kamari, G., Phitos, D., Snogerup, B. & Snogerup, S. Flora and vegetation of Yioura, N Sporades, Greece. *Willdenowia* **17**, 59-85 (1988).

Kelly, L. The vascular flora of Huggins Island, Onslow County, North Carolina. *Castanea* **71**, 295-311 (2006).

Kerguelen, M. *Base de Données Nomenclaturales de la Flore de France.* (2005) Available at: http://bbock.free.fr/botanica/BDNFF.php. (Date of access: 13 January 2012).

Kingston, N., Waldren, S. & Bradley, U. The phytogeographical affinities of the Pitcairn Islands – a model for south-eastern Polynesia? *J. Biogeogr.* **30**, 1311-1328 (2003).

Kirchner, F., Picot, F., Merceron, E. & Gigot, G. *Flore vasculaire de La Réunion*. (Conservatoire Botanique National de Mascarin, 2010).

Kissling, W. D. *et al.* Cenozoic imprints on the phylogenetic structure of palm species assemblages worldwide. *Proc. Natl. Acad. Sci. U. S. A.* **109**, 7379-7384 (2012).

Klotzenburg, M. *Checkliste der Gefässpflanzen Irlands.* (2011) Available at: http://www.saxifraga.de/eire/irl_artenlisten_gp.html. (Date of access: 4 April 2011).

Kristinsson, H. *Checklist of the vascular plants of Iceland*. (Náttúrufræðistofnun Íslands, 2008).

Lester-Garland, L. V. *A flora of the islands of Jersey: with a list of the plants of the Channel Islands in general, and remarks upon their distribution and geographical affinities*. (West, Newman & Co, 1903).

Levin, G. A. & Moran, R. The vascular flora of Socorro, Mexico. *Memoirs of the San Diego Society of Natural History* **16**, 1-71 (1989).

Marquand, E. D. *Flora of Guernsey and the lesser Channel Islands: namely Alderney, Sark, Herm, Jethou, and the adjacent islets*. (Dulau & Co, 1901).

Marticorena, C., Stuessy, T. F. & Baeza, C. M. Catalogue of the vascular flora of the Robinson Crusoe or Juan Fernández islands, Chile. *Gayana Bot.* **55**, 187-211 (1998).

McClatchey, W., Thaman, R. & Vodonaivalu, S. A preliminary checklist of the flora of Rotuma with Rotuman names. *Pac. Sci.* **54**, 345-363 (2000).

McCrea, J. *Inventory of the land conservation values of the Houtman Abrolhos Islands*. Vol. 151 147 (Department of Fisheries, Government of Western Australia, 2003).

Miller, A. G. & Morris, M. *Ethnoflora of the Soqotra Archipelago*. (Royal Botanic Garden, 2004).

Moran, R. *The flora of Guadalupe Island, Mexico*. (California Academy of Sciences, 1996).

Morat, P. *et al.* The taxonomic reference base Florical and characteristics of the native vascular flora of New Caledonia. *Adansonia* **34**, 179-221 (2012).

Nakamura, K., Suwa, R., Denda, T. & Yokota, M. Geohistorical and current environmental influences on floristic differentiation in the Ryukyu Archipelago, Japan. *J. Biogeogr.* **36**, 919-928 (2009).

New Zealand Plant Conservation Network. *New Zealand's Flora.* (2011) Available at: http://nzpcn.org.nz/page.asp?flora. (Date of access: 18 March 2011).

Parris, B. S. & Latiff, A. Towards a pteridophyte flora of Malaysia: A provisional checklist of taxa. *Malay. Nat. J.* **50**, 235-280 (1997).

Proctor, G. R. Checklist of the plants of Little Cayman. Geography and ecology of Little Cayman. *Atoll Res. Bull.* **241**, 71-80 (1980).

Proctor, G. R. *Ferns of Jamaica: a guide to the Pteridophytes*. Vol. 3 631 (British Museum (Natural History), 1985).

Proctor, G. R. *Ferns of Puerto Rico and the Virgin Islands*. Vol. 53 389 (Memoirs of the New York Botanical Garden, 1989).

Jahn, R. & Schönfelder, P. *Exkursionsflora für Kreta*. (Ulmer (Eugen), 1995).

Raulerson, L. Checklist of plants of the Mariana Islands. *University of Guam Herbarium Contribution* **37**, 1-69 (2006).

Renvoize, S. A. A floristic analysis of the western Indian Ocean coral islands. *Kew Bull.* **30**, 133-152 (1975).

Robinson, A. C., Canty, P. D., Wace, N. M. & Barker, R. M. The encounter 2002 expedition to the isles of St Francis, South Australia: flora and vegetation. *Trans. R. Soc. S. Aust.* **127**, 107-128 (2003).

Robinson, A. C., Canty, P. D. & Fotheringham, D. Investigator group expedition 2006: flora and vegetation. *Trans. R. Soc. S. Aust.* **132**, 173-220 (2008).

Roux, J. P. *Synopsis of the Lycopodiophyta and Pteridophyta of Africa, Madagascar and neighbouring islands*. (South African National Biodiversity Institute, 2009).

Sachet, M.-H. Flora and vegetation of Clipperton Island. *Proc. Calif. Acad. Sci.* **31**, 249-307 (1962).

Sandbakk, B. E., Alsos, I. G., Arnesen, G. & Elven, R. *The flora of Svalbard.* (1996) Available at: http://svalbardflora.net/. (Date of access: 16 March 2011).

Searle, J. & Madden, S. *Flora assessment of South Stradbroke Island*. (Gold Coast City Council, 2006).

Shaw, J. D., Spear, D., Greve, M. & Chown, S. L. Taxonomic homogenization and differentiation across Southern Ocean Islands differ among insects and vascular plants. *J. Biogeogr.* **37**, 217-228 (2010).

Stace, C. A., Ellis, R. G., Kent, D. H. & McCosh, D. J. *Vice-county Census Catalogue of the vascular plants of Great Britain, the Isle of Man and the Channel Islands*. (Botanical Society of the British Isles, 2003).

Stalter, R. & Lamont, E. E. The historical and extant flora of Sable Island, Nova Scotia, Canada. *J. Torrey Bot. Soc.* **133**, 362-374 (2006).

Stoddart, D. R. & Fosberg, F. R. Flora of the Phoenix Islands, central Pacific. *Atoll Res. Bull.* **393**, 1-60 (1994).

Sykes, W. R. Contributions to the flora of Niue. *Bulletin. Department of Scientific and Industrial Research, New Zealand* **200**, 321 (1970).

Takahashi, H., Barkalov, V. Y., Gage, S. & Zhuravlev, Y. N. A preliminary study of the flora of Chirpoi, Kuril Islands. *Acta Phytotaxon. Geobot.* **48**, 31-42 (1997).

Takahashi, H. *et al.* A preliminary checklist of the vascular plants of Chirinkotan, Kuril Islands. *Journal of Phytogeography and Taxonomy* **47**, 131-137 (1999).

Takahashi, H. *et al.* A floristic study of the vascular plants of Raikoke, Kuril Islands. *Acta Phytotaxon. Geobot.* **53**, 17-33 (2002).

Takahashi, H. *et al.* A floristic study of the vascular plants of Kharimkotan, Kuril Islands. *Bulletin of the Hokkaido University Museum* **3**, 41-66 (2006).

Tatewaki, M. Geobotanical studies on the Kurile Islands. *Acta Horti Gotoburgensis* **21**, 43-123 (1957).

Taylor, R. *Straight through from London: the Antipodes and Bounty Islands, New Zealand*. (Heritage Expeditions New Zealand, 2006).

Telford, I. R. H. *Cocos (Keeling) Islands: Species lists.* (1993) Available at: http://www.environment.gov.au/biodiversity/abrs/online-resources/flora/50/index.html. (Date of access: 7 April 2011).

Thaman, R. R., Fosberg, F. R., Manner, H. I. & Hassall, D. C. The flora of Nauru. *Atoll Res. Bull.* **392**, 1-233 (1994).

The Chinese Academy of Sciences. *Chinese Virtual Herbarium.* (2009) Available at: http://www.cvh.org.cn/. (Date of access: 23 June 2011).

UIB. *Herbario virtual del Mediterráneo Occidental.* (2007) Available at: http://herbarivirtual.uib.es/cas-med/index.html. (Date of access: 7 August 2012).

University of Kent. *Cook Islands Biodiversity and Ethnobiology Database.* (2012) Available at: http://cookislands.pacificbiodiversity.net/cibed/dbs/search.html. (Date of access: 12 April 2012).

Wace, N. M. The vegetation of Gough Island. *Ecol. Monogr.* **31**, 337-367 (1961).

Wace, N. & Dickson, J. H. The terrestrial botany of the Tristan da Cunha Islands. *Philos. Trans. R. Soc. Lond. B Biol. Sci.* **249**, 273-360 (1965).

Wagner, W. L. & Lorence, D. H. *Flora of the Marquesas Islands website.* (2002) Available at: http://botany.si.edu/pacificislandbiodiversity/marquesasflora/index.htm. (Date of access: 16 December 2010).

Wagner, W. L., Herbst, D. R. & Lorence, D. H. *Flora of the Hawaiian Islands website.* (2005) Available at: http://botany.si.edu/pacificislandbiodiversity/hawaiianflora/index.htm. (Date of access: 16 October 2010).

Wellington Botanical Society. *Native vascular plants of Great Barrier Island*. (Wellington Botanical Society, 2008).

Whistler, W. A. Vegetation and flora of the Aleipata Islands, Western Samoa. *Pac. Sci.* **37**, 227-249 (1983).

Whistler, W. A. Botanical survey of Diego Garcia, Chagos Archipelago, British Indian Ocean Territory. *Isle Botanica* **Appendix E1**, 1-72 (1996).

Whistler, W. A. *A study of the rare plants of American Samoa*. (US Fish and Wildlife Service, 1998).

Supplementary References S2. References cited in this supplement.

1 Willson, M. F. & Traveset, A. in *Seeds: the ecology of regeneration in plant communities* Vol. 2 (ed M. Fenner) 85-107 (CABI, 2000).

2 Westoby, M., Leishman, M., Lord, J., Poorter, H. & Schoen, D. J. Comparative ecology of seed size and dispersal [and discussion]. *Philos. Trans. R. Soc. Lond. B Biol. Sci.* **351**, 1309-1318 (1996).

3 Moles, A. T. *et al.* A brief history of seed size. *Science* **307**, 576-580 (2005).

4 Cavender-Bares, J., Kozak, K. H., Fine, P. V. A. & Kembel, S. W. The merging of community ecology and phylogenetic biology. *Ecol. Lett.* **12**, 693-715 (2009).

5 Crisp, M. D. *et al.* Phylogenetic biome conservatism on a global scale. *Nature* **458**, 754-756 (2009).

6 Donoghue, M. J. A phylogenetic perspective on the distribution of plant diversity. *Proc. Natl. Acad. Sci. U. S. A.* **105**, 11549-11555 (2008).

7 Givnish, T. J. Ecology of plant speciation. *Taxon* **59**, 1326-1366 (2010).

8 Rakotoarinivo, M. *et al.* Palaeo-precipitation is a major determinant of palm species richness patterns across Madagascar: a tropical biodiversity hotspot. *Proc. R. Soc. Biol. Sci. Ser. B* **280**, 20123048 (2013).

9 Morat, P. Our knowledge of the flora of New Caledonia: endemism and diversity in relation to vegetation types and substrates. *Biodiv. Lett.* **1**, 72-81 (1993).

10 Kissling, W. D. *et al.* Cenozoic imprints on the phylogenetic structure of palm species assemblages worldwide. *Proc. Natl. Acad. Sci. U. S. A.* **109**, 7379-7384 (2012).

11 Eiserhardt, W. L., Svenning, J.-C., Kissling, W. D. & Balslev, H. Geographical ecology of the palms (Arecaceae): determinants of diversity and distributions across spatial scales. *Ann. Bot. (Lond.)* **108**, 1391-1416 (2011).

12 Kissling, W. D. *et al.* Quaternary and pre-Quaternary historical legacies in the global distribution of a major tropical plant lineage. *Glob. Ecol. Biogeogr.* **21**, 909-921 (2012).

13 Kreft, H., Sommer, J. H. & Barthlott, W. The significance of geographic range size for spatial diversity patterns in Neotropical palms. *Ecography* **29**, 21-30 (2006).

14 Tomlinson, P. B. The uniqueness of palms. *Bot. J. Linn. Soc.* **151**, 5-14 (2006).

15 Savolainen, V. *et al.* Sympatric speciation in palms on an oceanic island. *Nature* **441**, 210-213 (2006).

16 Tryon, R. Development and evolution of fern floras of Oceanic Islands. *Biotropica* **2**, 76-84 (1970).

17 Kisel, Y. & Barraclough, T. G. Speciation has a spatial scale that depends on levels of gene flow. *Am. Nat.* **175**, 316-334 (2010).

18 Hennequin, S., Kessler, M., Lindsay, S. & Schneider, H. Evolutionary patterns in the assembly of fern diversity on the oceanic Mascarene Islands. *J. Biogeogr.* **41**, 1651-1663 (2014).

19 Smith, A. R. Comparison of fern and flowering plant distributions with some evolutionary interpretations for ferns. *Biotropica* **4**, 4-9 (1972).

20 Kreft, H., Jetz, W., Mutke, J. & Barthlott, W. Contrasting environmental and regional effects on global pteridophyte and seed plant diversity. *Ecography* **33**, 408-419 (2010).

21 Schuettpelz, E., Schneider, H., Huiet, L., Windham, M. D. & Pryer, K. M. A molecular phylogeny of the fern family Pteridaceae: Assessing overall relationships and the affinities of previously unsampled genera. *Mol. Phylogenet. Evol.* **44**, 1172-1185 (2007).

22 Brodribb, T. J. & McAdam, S. A. M. Passive origins of stomatal control in vascular plants. *Science* **331**, 582-585 (2011).

23 Mabberley, D. J. *Mabberley's plant-book: a portable dictionary of plants, their classification and uses*. (Cambridge University Press, 2008).

24 The Angiosperm Phylogeny Group. An update of the Angiosperm Phylogeny Group classification for the orders and families of flowering plants: APG III. *Bot. J. Linn. Soc.* **161**, 105-121 (2009).

25 Bell, C. D., Soltis, D. E. & Soltis, P. S. The age and diversification of the angiosperms re-revisited. *Am. J. Bot.* **97**, 1296-1303 (2010).

26 Davies, T. J. *et al.* Darwin's abominable mystery: insights from a supertree of the angiosperms. *Proc. Natl. Acad. Sci. U. S. A.* **101**, 1904-1909 (2004).

27 Stevens, P. F. *Angiosperm Phylogeny Website. Version 12.* (2012) Available at: http://www.mobot.org/MOBOT/research/APweb/. (Date of access: 1 May 2013).

28 Baker, W. J. & Couvreur, T. L. P. Global biogeography and diversification of palms sheds light on the evolution of tropical lineages. I. Historical biogeography. *J. Biogeogr.* **40**, 274-285 (2013).

29 Lehtonen, S. Towards resolving the complete fern tree of life. *PLoS One* **6**, e24851 (2011).

30 Drummond, A. J., Suchard, M. A., Xie, D. & Rambaut, A. Bayesian phylogenetics with BEAUti and the BEAST 1.7. *Mol. Biol. Evol.* **29**, 1969-1973 (2012).

31 Ott, M., Zola, J., Stamatakis, A. & Aluru, S. in *Proceedings of the 2007 ACM/IEEE conference on Supercomputing* 1-11 (ACM, Reno, NV, 2007).

32 Stamatakis, A. RAxML-VI-HPC: maximum likelihood-based phylogenetic analyses with thousands of taxa and mixed models. *Bioinformatics* **22**, 2688-2690 (2006).

33 Christenhusz, M., Zhang, X.-C. & Schneider, H. A linear sequence of extant families and genera of lycophytes and ferns. *Phytotaxa* **19**, 7-54 (2011).

34 Schneider, H. *et al.* Ferns diversified in the shadow of angiosperms. *Nature* **428**, 553-557 (2004).

35 Jønsson, K. A. *et al.* Ecological and evolutionary determinants for the adaptive radiation of the Madagascan vangas. *Proc. Natl. Acad. Sci. U. S. A.* **109**, 6620-6625 (2012).

36 Price, J. P. & Wagner, W. L. Speciation in Hawaiian angiosperm lineages: cause, consequence, and mode. *Evolution* **58**, 2185-2200 (2004).

37 Emerson, B. C. & Gillespie, R. G. Phylogenetic analysis of community assembly and structure over space and time. *Trends Ecol. Evol.* **23**, 619-630 (2008).

38 Wiens, J. J. & Donoghue, M. J. Historical biogeography, ecology and species richness. *Trends Ecol. Evol.* **19**, 639-644 (2004).

39 Paradis, E., Claude, J. & Strimmer, K. ape: analyses of phylogenetics and evolution in R language. *Bioinformatics* **20**, 289-290 (2004).

40 Kembel, S. W. *et al.* Picante: R tools for integrating phylogenies and ecology. *Bioinformatics* **26**, 1463-1464 (2010).

41 Diniz-Filho, J. A. F. & Bini, L. M. Modelling geographical patterns in species richness using eigenvector-based spatial filters. *Glob. Ecol. Biogeogr.* **14**, 177-185 (2005).

42 Griffith, D. A. & Peres-Neto, P. R. Spatial modeling in ecology: the flexibility of eigenfunction spatial analyses. *Ecology* **87**, 2603-2613 (2006).

43 Wood, S. N. & Augustin, N. H. GAMs with integrated model selection using penalized regression splines and applications to environmental modelling. *Ecol. Model.* **157**, 157-177 (2002).

44 Barton, K. MuMIn: Multi-model inference v. 1.9.5 (Package for R statistical software, 2013).

45 Oksanen, J. *et al.* vegan: community ecology package v. 2.0-7 (Package for R statistical software, 2013).

46 Bivand, R. *et al.* spdep: spatial dependence: weighting schemes, statistics and models v. 0.5-56 (Package for R statistical software, 2011).

47 Dutilleul, P., Clifford, P., Richardson, S. & Hemon, D. Modifying the t Test for assessing the correlation between two spatial processes. *Biometrics* **49**, 305-314 (1993).

48 Takhtajan, A. *Floristic regions of the world*. (University of California Press, 1986).

49 Graves, G. R. & Gotelli, N. J. Neotropical land-bridge avifaunas: new approaches to null hypotheses in biogeography. *Oikos* **41**, 322-333 (1983).

50 R Development Core Team. R: a language and environment for statistical computing (R Foundation for Statistical Computing, Vienna, Austria, 2013).
